# Visual activity enhances neuronal excitability in thalamic relay neurons

**DOI:** 10.1126/sciadv.adp4627

**Published:** 2025-01-22

**Authors:** Maël Duménieu, Laure Fronzaroli-Molinieres, Loïs Naudin, Cécile Iborra-Bonnaure, Anushka Wakade, Emilie Zanin, Aurore Aziz, Norbert Ankri, Salvatore Incontro, Danièle Denis, Béatrice Marquèze-Pouey, Romain Brette, Dominique Debanne, Michaël Russier

**Affiliations:** ^1^ Aix-Marseille Université, INSERM, UNIS, Marseille, France.; ^2^ Sorbonne Université, INSERM, CNRS, Institut de la Vision, Paris, France.; ^3^ Department of Ophthalmology, CHU Nord, Marseille, France.

## Abstract

Amblyopia, a highly prevalent loss of visual acuity, is classically thought to result from cortical plasticity. The dorsal lateral geniculate nucleus (dLGN) has long been held to act as a passive relay for visual information, but recent findings suggest a largely underestimated functional plasticity in the dLGN. However, the cellular mechanisms supporting this plasticity have not yet been explored. We show here that monocular deprivation (MD), an experimental model of amblyopia, reduces the intrinsic excitability of dLGN cells. Furthermore, dLGN neurons exhibit long-term potentiation of their intrinsic excitability (LTP-IE) when suprathreshold afferent retinal inputs are stimulated at 40 hertz or when spikes are induced with current injection. LTP-IE is observed after eye opening, requires calcium influx, is expressed through the down-regulation of Kv1 channels, and is altered following MD. In conclusion, our study provides the first evidence for intrinsic plasticity in dLGN neurons induced by natural stimuli.

## Teaser

Visual thalamic neurons express long-lasting changes in intrinsic excitability during amblyopia and following retinal stimulation.

## INTRODUCTION

Activity-dependent plasticity in the visual system is classically thought to be exclusively expressed at the cortical level whereas the dorsal lateral geniculate nucleus (dLGN), a primary recipient structure of retinal inputs at the thalamic level, is traditionally considered to be just a relay of visual information. Monocular deprivation, an experimental model of amblyopia, has been thought for a long time to produce no effect on the receptive field properties of dLGN neurons (*1*). However, this pioneering conclusion has been challenged by later works indicating that this simplistic view does not hold (*2*–*4*). The spatial resolution of dLGN neurons activated by the deviate eye in kittens reared with a squint is considerably reduced compared to that of neurons activated by the normal eye (*5*). In amblyopic patients, functional deficits in visual responses are already observed at the stage of the LGN (*6*). Moreover, in contrast to what was previously established, about half of dLGN relay neurons in a given monocular territory receive inputs from both eyes, indicating a potential binocularity for a large proportion of dLGN neurons (*7*–*9*). In addition, monocular deprivation (MD) was shown to produce a large shift in ocular dominance in dLGN neurons (*3*, *10*, *11*). However, the cellular mechanisms underlying functional changes occurring in these in vivo studies remain unclear. In particular, the precise locus of the plasticity has not been clearly identified.

Among the cellular mechanisms that may occur during functional plasticity, the regulation of intrinsic neuronal excitability is a potential candidate. Many brain regions including visual areas express intrinsic plasticity (*12*, *13*). Long-term intrinsic plasticity has been reported in central neurons following stimulation of afferent glutamate inputs (*14*), spiking activity (*15*), sensory stimulation (*16*), or following sensory deprivation (*17*, *18*). However, it is not yet known whether dLGN relay cells also express intrinsic plasticity.

We show here that MD reduces intrinsic excitability of dLGN neurons. In addition, we report the induction of long-term potentiation of intrinsic excitability (LTP-IE) in dLGN relay neurons following stimulation of the retinal afferent inputs that evoke spiking activity. This enhanced excitability is observed in a specific developmental window (i.e., after eye opening). LTP-IE is mediated by the down-regulation of Kv1 channels through the activation of cAMP-dependent protein kinase (PKA). Our results thus deeply modify the current view regarding the involvement of the thalamic stage in amblyopia by demonstrating unambiguously the existence of activity-dependent plasticity of intrinsic neuronal excitability in dLGN relay neurons.

## RESULTS

### MD reduces intrinsic excitability in dLGN neurons

**Fig. 1.**
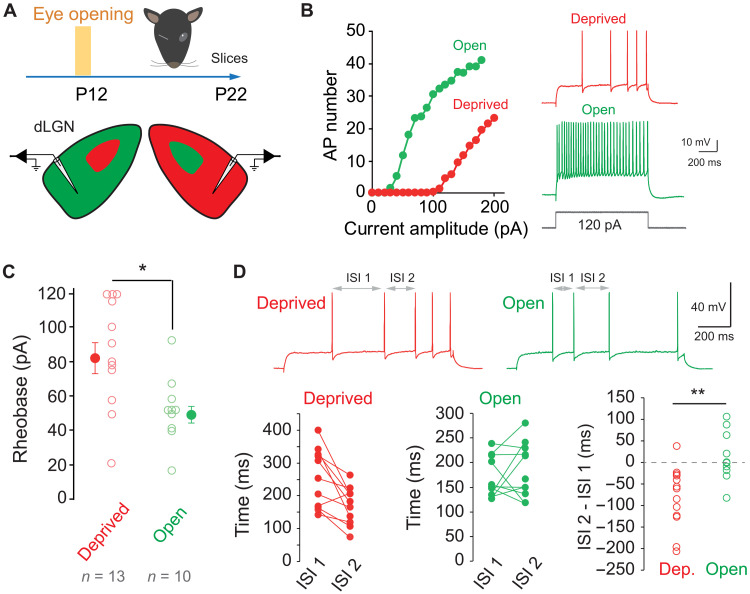
Monocular deprivation reduces neuronal excitability in dLGN relay cells. (**A**) Scheme of the experimental protocol. Lids on one eye were sutured in Long Evans rats at P12 (i.e., just before eye opening). Animals were kept until P22, and acute slices containing both dLGN were prepared. (**B**) Left: Comparison of input-output curves from neurons recorded in the contralateral segments of the dLGN activated by the open (green) and deprived (red) eye. Note the difference in rheobase and in intrinsic excitability. Right: Comparison of the firing in response to the same current in the two cells. (**C**) Comparison of the rheobase in deprived and open dLGN neurons. Mann-Whitney test, *P < 0.05. (**D**) Top: Traces from neurons activated by the deprived (red) and open (green) eye to study the adaptation of the first three spikes. Bottom left: In neurons activated by the deprived eye, a clear reduction of ISI 2 compared to ISI 1 is observed. Middle: In neurons activated by open eye, no change is observed. Right: Comparison of the difference in ISI for neurons activated by the deprived and open eye. Mann-Whitney test, **P < 0.01.

To determine the effects of MD on neuronal excitability at the thalamic stage, Longs Evans rats were monocularly deprived just before eye opening [i.e., at postnatal day 12 (P12)] during 10 days, and acute slices containing the dLGN were obtained (Fig. 1A). Contralateral projection zones were identified by the injection of cholera toxin conjugated with Alexa Fluor 594 and Alexa Fluor 488 in separate experiments (fig. S1A). Relay cells were recorded in whole-cell configuration from the monocular segment of the contralateral projection zone to the closed eye or from the monocular segment of the contralateral projection zone to the open eye of the dLGN. Compared to neurons activated by the open eye, neurons activated by the deprived eye were less excitable and exhibited a higher rheobase when the neuron was held at a membrane potential of −56 mV to inactivate the T-type calcium current (fig. S1B) (deprived side: 82 ± 6 pA, *n* = 13 cells in 10 rats versus open side: 51 ± 7 pA, *n* = 10 cells in nine rats; Mann-Whitney test, *P* < 0.05; Fig. 1, B and C, and fig. S1, C and D). No significant change in the spike threshold (deprived side: −40.1 ± 1.0, *n* = 13 versus −38 ± 0.8 mV, *n* = 10 on the open side, Mann-Whitney test, *P* > 0.1; fig. S1E), holding current (deprived side: 30 ± 6 pA, *n* = 13 versus 18 ± 6 pA, *n* = 10 on the open side; Mann-Whitney *P* > 0.5), or in input resistance (deprived side: 441 ± 69 megohm, *n* = 13; open side: 548 ± 46 megohm, *n* = 10, Mann-Whitney test, *P* > 0.1; fig. S1E) was observed between deprived and spared neurons. Nevertheless, the temporal pattern of the spike discharge was found to be different in deprived and spared neurons. While deprived neurons displayed a reduction in the second interspike interval (ISI 2) compared to the first (i.e., ISI 1), neurons activated by the open eye did not show this reduction (deprived side: −83.4 ± 19.8 ms, *n* = 13 versus open side: 13.5 ± 18.1, *n* = 10; Mann-Whitney, *P* < 0.01; Fig. 1D). This difference was due to the ramp measured on subthreshold traces (fig. S1F). Our results strongly suggest that visual activity promotes neuronal excitability, while visual deprivation reduces intrinsic excitability in dLGN neurons.

### Induction of LTP-IE in dLGN relay cells

**Fig. 2.**
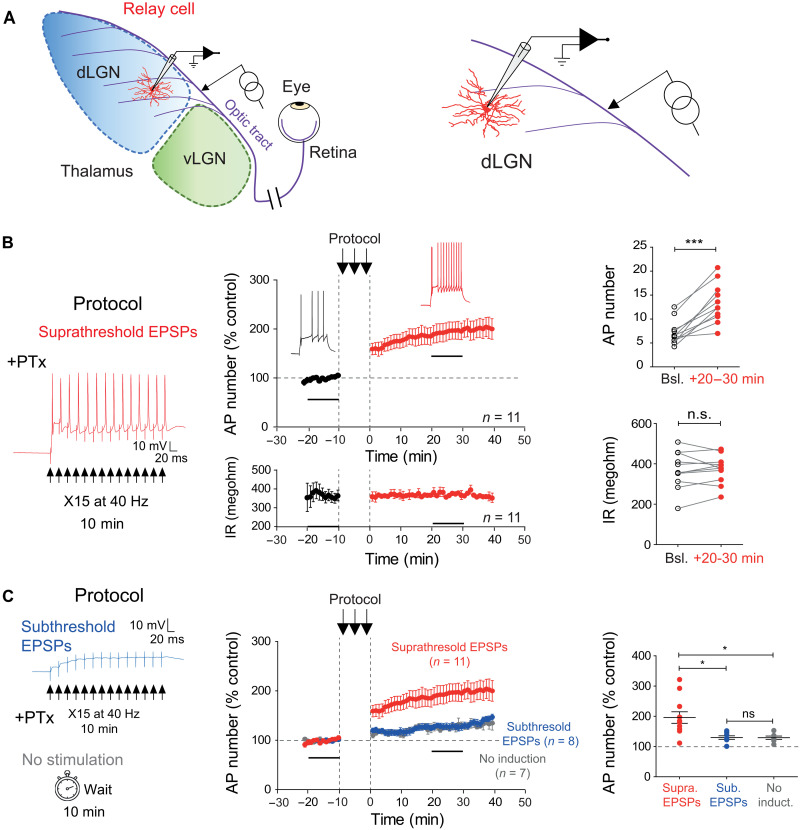
Synaptic induction of LTP-IE in dLGN relay neurons. (**A**) Recording configuration of relay cells in the dLGN. Left: Scheme of the LGN with the dorsal LGN (dLGN, in blue), the ventral LGN (vLGN, green), and the afferent axons from the retina (optic tract, violet). Right: Recording and stimulation arrangements. (**B**) Trains of suprathreshold EPSPs delivered at a frequency of 40 Hz induce LTP-IE in dLGN relay neurons (membrane potential: −65 mV). Left: Protocol. Middle: Time course of the spike number normalized to the control period (top) and time course of the input resistance (Rin, bottom). Right: Comparison of the AP number (top) and the Rin (bottom). Wilcoxon test, ***P < 0.001; ns, not significant. (**C**) Trains of subthreshold EPSPs do not induce LTP-IE. Left: Protocol of stimulation with subthreshold EPSPs (top) and without any stimulation (bottom). Middle: Time courses of excitability changes following stimulation with subthreshold EPSPs (blue) and no stimulation (gray) compared to the time-course of excitability changes following stimulation with suprathreshold EPSPs. Right: Comparison of excitability changes in dLGN neurons following suprathreshold EPSPs (red), subthreshold EPSPs (blue), and no stimulation (gray). Mann-Whitney test, *P < 0.05.

We next checked whether activation of retinal inputs could induce plasticity of intrinsic neuronal excitability during the recording of a dLGN relay neuron. Whole-cell recordings from dLGN relay cells were obtained in acute slices from young rats (P19 to P25) at a membrane potential of −65 mV, and a stimulating electrode was placed on the optic tract to evoke an excitatory post-synaptic potential (EPSP; Fig. 2A). GABA_A_ receptors were blocked with picrotoxin (100 μm). EPSP amplitude increased with stimulus intensity and evoked an action potential (AP, fig. S2A). LTP-IE measured 20 to 30 min after the stimulation was induced in dLGN relay cells following stimulating suprathreshold EPSP (i.e., producing an AP on the top of each EPSP) at a frequency of 40 Hz during 10 min. The number of spikes evoked by an identical pulse of current increased by a factor two (196 ± 19%, *n* = 11; mean spike number: 7.1 ± 0.8 before and 13.3 ± 1.2 after stimulation, Wilcoxon test, *P* < 0.001; Fig. 2B). This plasticity was not associated with any change in input resistance (103 ± 1%, *n* = 11; mean resistance: 369 ± 28 megohm before and 373 ± 21 megohm after stimulation, Wilcoxon test, *P* > 0.5; Fig. 2B and fig. S2C), nor in holding current to maintain the membrane potential at −65 mV (fig. S2B). The presence of spikes during the induction was found to be critical in the magnitude of LTP-IE. When subthreshold EPSPs were evoked, the magnitude of LTP-IE was significantly smaller (130 ± 6%, *n* = 8) than that produced by suprathreshold EPSPs eliciting APs (196 ± 19%, *n* = 11, Wilcoxon test, *P* < 0.05; Fig. 2C). Here again, input resistance was unchanged after stimulation of subthreshold EPSPs (fig. S2D). Furthermore, subthreshold EPSPs did not induce a larger LTP-IE than that observed when the stimulation was suspended during 10 min (129 ± 6%, *n* = 7, Mann-Whitney, *P* > 0.05; Fig. 2C), indicating that spiking activity is critical for inducing LTP-IE.

### AP firing is sufficient to induce LTP-IE in dLGN relay neurons

**Fig. 3.**
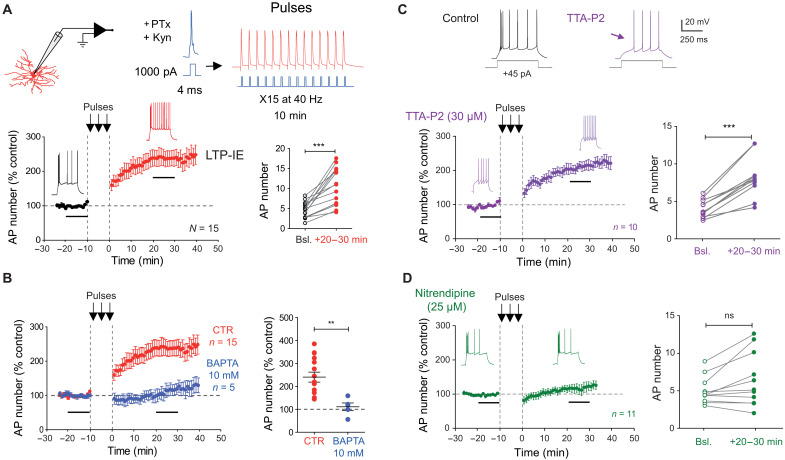
Induction of LTP-IE in dLGN relay neurons with spiking activity. (**A**) Trains of APs at 40 Hz induce LTP-IE in dLGN relay neurons. Top: Recording configuration and protocol. Bottom left: Time course of neuronal excitability before and after trains of APs (membrane potential: −65 mV). Bottom right: Group data for AP number. Wilcoxon test, ***P < 0.001. Bsl, baseline; Kyn, kynurenate, PTx, picrotoxin. (**B**) Intracellular BAPTA blocks LTP-IE. Left: Time courses of changes in excitability in BAPTA (blue) compared to control (red) (membrane potential: −65 mV). Right: Comparison of the normalized change in neuronal excitability. Mann-Whitney test, **P < 0.01. (**C**) LTP-IE is not mediated by T-type calcium channels. The T-type calcium channel blocker TTA-P2 suppressed the initial burst and uncovered a ramp-and-delay phenotype (membrane potential: −65 mV). In the presence of TTA-P2, LTP-IE is still induced. Left: Time course. Right group data. Wilcoxon test, ***P < 0.001. (**D**) Lack of LTP-IE in the presence of the L-type calcium channel blocker, nitrendipine (membrane potential: −65 mV). Left: Time course. Right: Group data. Wilcoxon test, ns, *P* > 0.1.

To check whether spiking activity alone was the key trigger of LTP-IE in dLGN neurons, APs were induced by current pulses delivered at 40 Hz in the presence of ionotropic glutamate (kynurenate, 2 mM) and GABA_A_ receptors antagonists (picrotoxin, 100 μM). A twofold increase in excitability that could not be distinguished from that induced by synaptic stimulation was observed in these conditions (240 ± 22%, *n* = 15; mean spike number: 4.6 ± 0.5 before and 10.6 ± 1.2 after stimulation, Wilcoxon test, *P* < 0.001; Fig. 3A). *R*_in_ remained unchanged (396 ± 24 megohm before versus 395 ± 22 megohm after induction, *n* = 15, Wilcoxon test, *P* > 0.1). We conclude that spiking activity is critical for induction of LTP-IE in dLGN neurons.

We next evaluated whether the triggering signal of LTP-IE relied on an elevation of intracellular calcium. As APs activate voltage-gated calcium channels, we tested whether chelating intracellular calcium changes with 1,2-bis(2-aminophenoxy)ethane-*N*,*N*,*N*′,*N*′-tetraacetic acid (BAPTA) (10 mM) would prevent LTP-IE induction in dLGN neurons. In this condition, no LTP-IE was observed (111 ± 17%, *n* = 5; Fig. 3B). Given that dLGN relay cells express a large T-type calcium current (*19*, *20*), we first investigated the role of these channels in the induction of LTP-IE. Blockade of T-type channels with the specific Cav3.2 channel blocker, 3,5-dichloro-N-[1-(2,2-dimethyl-tetrahydro-pyran-4-ylmethyl)-4-fluoro-piperidin-4-ylmethyl]-benzamide (TTA-P2) (30 μM), abolished the depolarizing rebound and the initial burst of APs elicited with current injection (Fig. 3C) but left intact the increased neuronal excitability induced by spiking activity at 40 Hz (197 ± 11%, *n* = 10; mean spike number: 4.2 ± 0.4 before and 8.5 ± 0.9 after stimulation, Wilcoxon test, *P* < 0.01; Fig. 3C). These results indicate that calcium is required for induction of LTP-IE, but the source of calcium is not mediated by T-type Ca^2+^ channels. Thus, we checked whether L-type (Cav1) Ca^2+^ channels could be involved in LTP-IE induction. In the presence of the L-type Ca^2+^ channel blocker, nitrendipine (25 μM), no LTP-IE was induced by spiking activity (122 ± 11%, *n* = 11; AP number before: 5.0 ± 1.0 after: 6.4 ± 1.1, Wilcoxon test, *P* > 0.1; Fig. 3D), indicating that sodium APs activate L-type Ca^2+^ channels to trigger the calcium influx leading to LTP-IE.

### LTP-IE can only be induced after eye opening

**Fig. 4.**
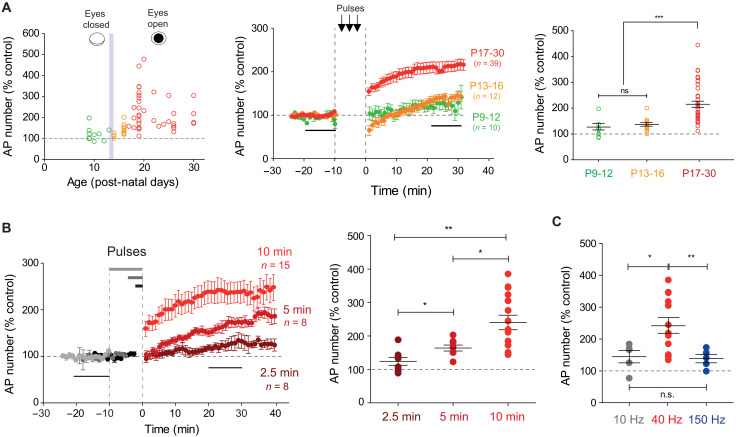
Developmental profile and incidence of stimulus duration and frequency on LTP-IE. (**A**) Left: Normalized LTP-IE as a function of the postnatal age. The vertical gray bar represents the time of eye opening. Note that before eye opening, no LTP-IE is induced. Middle: Time courses of LTP-IE induced at P9 to P12 (green), P13 to P16 (orange), and P17-P30 (red). Right: Comparison of LTP-IE at different ages. Mann-Whitney test, ***P < 0.001. (**B**) Role of the stimulus duration on LTP-IE in dLGN neurons. Left: Time course of LTP-IE induced by a stimulation lasting 2.5 min (brown), 5 min (dark red), and 10 min (red). Right: Comparison of the magnitude of LTP-IE. Mann-Whitney test, *P < 0.05 and **P < 0.01. (**C**) Frequency dependence of LTP-IE. Comparison of the magnitude of LTP-IE induced by stimulation at 10, 40, and 150 Hz. Mann-Whitney test, *P < 0.05 and **P < 0.01.

We next examined the developmental profile of LTP-IE induced by spiking stimulation at 40 Hz in dLGN neurons. For this, we extended the range of age from P19 to P25 to P9 to P30. While LTP-IE was robustly induced from P17 to P30 (215 ± 12%, *n* = 39; Fig. 4A), the magnitude of LTP-IE was much reduced before eye opening (P9 to P12; 127 ± 13%, *n* = 10; Fig. 4A) and just after eye opening (P13 to P16; 137 ± 7%, *n* = 12; Fig. 4A). In contrast to the situation at P17 to P30, the spike number was not significantly increased at P9 to P12 (from 3.0 ± 0.3 to 3.9 ± 0.6 spikes, *n* = 10; fig. S3A). Although little LTP-IE was observed at early age, the spiking profile was similar to that obtained in more mature animals (fig. S3B). These results show that LTP-IE is developmentally regulated as it is only robustly expressed after P17, i.e., 3 to 4 days after eye opening.

### Properties of LTP-IE induction in dLGN relay neurons

We next checked whether the duration of stimulation was critical for LTP-IE induction with 40-Hz stimulation by current injection. While no significant LTP-IE was induced with a stimulation time of 2.5 min (124 ± 10%, *n* = 8; Wilcoxon test, *P* > 0.1, Fig. 4B), a significant LTP-IE was induced with a stimulation time of 5 min (164 ± 9%, *n* = 8; Wilcoxon test, *P* < 0.01; Fig. 4B). Nevertheless, the magnitude of LTP-IE was significantly smaller than that produced by 10-min stimulation (MW, *P* < 0.05; Fig. 4B).

As dLGN neurons are able to fire over a wide range of frequencies during visual stimulation in rodents, we checked the effect of stimulation frequency on intrinsic excitability. At 10 Hz for 10 min, no significant facilitation was observed (145 ± 9%, *n* = 5; Fig. 4C; mean spike number, 5.3 ± 0.7, *n* = 5 before 10-Hz stimulation, and 7.4 ± 0.9 after 10-Hz stimulation, Wilcoxon test, *P* > 0.05; fig. S3C). Similar results were obtained at 150 Hz (Fig. 4C). We conclude that LTP-IE in dLGN neurons is frequency selective and is preferentially induced by stimulation at 40 Hz.

### Expression of LTP-IE in dLGN neurons involves Kv1 channels

**Fig. 5.**
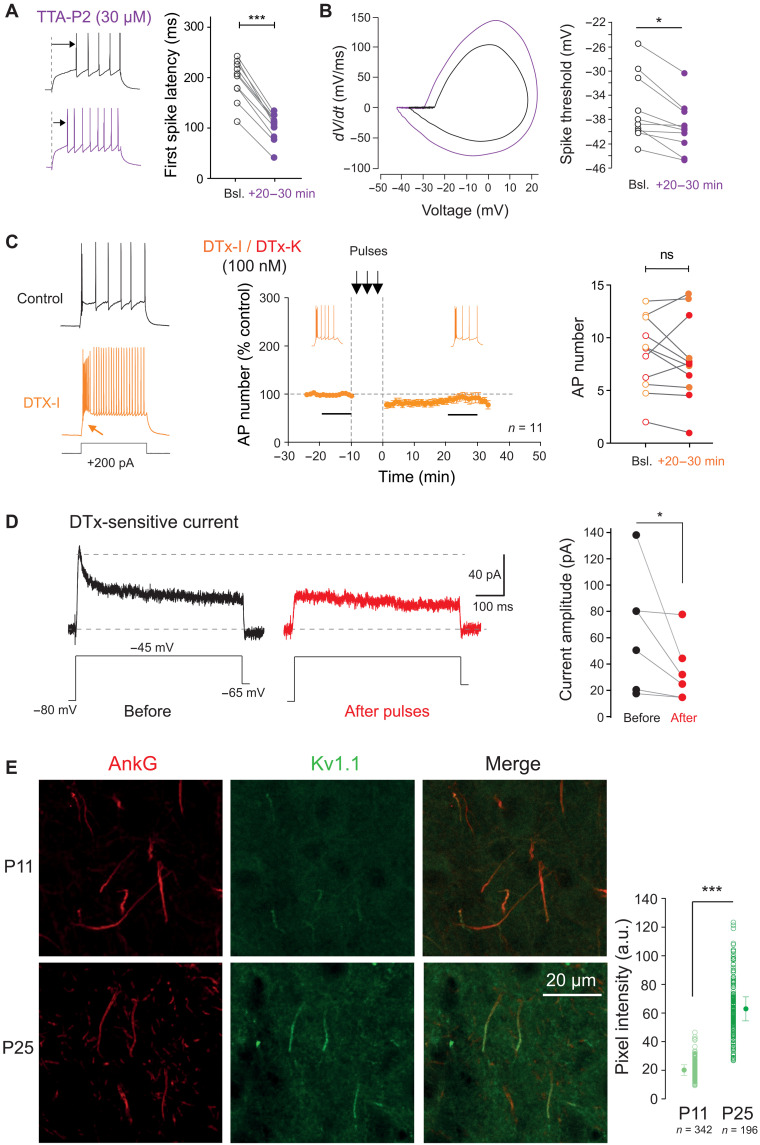
Expression mechanisms of LTP-IE in dLGN relay neurons: Kv1 channels. (**A**) Reduction of the first spike latency in dLGN neurons following induction of LTP-IE in the presence of T-type channel blocker. Left: Comparison of the first spike latency before and after induction of LTP-IE in the presence of TTA-P2. Right: Group data. Wilcoxon test, ***P < 0.001. (**B**) Hyperpolarization of the AP threshold. Left: Phase plots. Right: Pooled data. Wilcoxon test, *P < 0.05. (**C**) Pharmacological blockade of Kv1 channels with DTx-I/K prevents the induction of LTP-IE by 40-Hz spiking activity. Left: Effect of DTx-I on a dLGN relay neuron. Middle: Time course of the LTP-IE in the presence of DTx-I or DTx-K. Right: Group data. Orange DTx-I, red DTx-K. Wilcoxon test. (**D**) DTx-sensitive current before and after induction of LTP-IE with. Left: Representative traces. Right: Group data. Wilcoxon test, *P < 0.05. (**E**) Kv1.1 channels are immuno-detected on the AIS of dLGN neurons at P25 (bottom raw) but not at P11 (top raw). Left: Column, ankyrin G (AnkG) labeling. Middle column: Kv1.1 immunolabeling. Right: Column, merge of the Kv1.1 and AnkG immunolabeling. Right: Pooled data. Mann-Whitney test, ***P < 0.001.

We next determined the expression mechanisms of LTP-IE in dLGN neurons. In the presence of the Cav3.2 channel blocker, TTA-P2, the initial burst disappeared and revealed a ramp-and-delay phenotype that is characteristic of the contribution of Kv1 channels in control cells (*21*). Furthermore, under Cav3.2 channel blockade, the induction of LTP-IE was associated with a reduction in the first spike latency (50 ± 1%, *n* = 9; mean latency: 187 ± 4 ms before and 96 ± 3 ms after stimulation, Wilcoxon test, *P* < 0.01; Fig. 5A). Similar changes in the latency of the first spike after the initial T-type calcium burst were also observed in LTP-IE induced either by suprathreshold synaptic stimulation (50 ± 4%, *n* = 10; mean latency: 184 ± 18 ms before and 92 ± 12 ms after stimulation, Wilcoxon test, *P* < 0.01; fig. S4A) or by trains of APs induced by direct current injection (63 ± 4%, *n* = 15; mean latency: 274 ± 22 ms before and 174 ± 21 ms after stimulation, Wilcoxon test, *P* < 0.01; fig. S4B). In addition, the jitter to the first spike was found to be reduced following LTP-IE induction in the presence pf TTA-P2 (SE: 38.6 ± 4.2 ms before induction versus 14.4 ± 1.1 ms, *n* = 10, Wilcoxon, *P* < 0.01; fig. S5), and the spike threshold was lowered (from −36.1 ± 1.7 mV to −39.4 ± 1.3 mV, *n* = 10, Wilcoxon test, *P* < 0.01; Fig. 5B). Lower spike threshold was also seen following induction of LTP-IE with suprathreshold retinal inputs (from −37.4 ± 0.8 mV to −39.2 ± 0.8 mV, *n* = 10; Wilcoxon test, *P* < 0.01, fig. S6A) or current injection (from −39.6 ± 0.9 mV to −42.8 ± 1.1 mV, *n* = 15; Wilcoxon test, *P* < 0.001; fig. S6B). These modulations in first spike latency, jitter, and threshold are indicative of Kv1 channel down-regulation (*21*–*23*). To verify this hypothesis, experiments were conducted in the presence of the broad spectrum Kv1 channel blocker, DTx-I, or in the presence of the specific Kv1.1 channel blocker, DTx-K. As expected, DTx-I (100 nM) or DTx-K (100 nM) was found to mimic LTP-IE (i.e., increase of AP number by a factor ~ 3, AP hyperpolarization by ~4 mV, and latency of the first spike after the burst by half; Fig. 5C and fig. S7), but LTP-IE was totally occluded in the presence of DTx-I or DTx-K (94 ± 8%, *n* = 11; mean spike number: 8.4 ± 1.0 before and 8.0 ± 1.2 after stimulation; Wilcoxon test, *P* > 0.1; Fig. 5C). To confirm the reduction of Kv1 activity, the DTx-sensitive current was recorded before and after induction of LTP-IE with current pulses. A clear reduction of the amplitude of the DTx-sensitive current was observed (65 ± 8 before and 35 ± 4 pA after, *n* = 6, Wilcoxon test, *P* < 0.05; Fig. 5D). We conclude that LTP-IE in dLGN relay neurons is mediated by the down-regulation of Kv1 channels.

As LTP-IE induction requires Kv1 channels and LTP-IE is absent before eye opening, we hypothesized that Kv1 channel expression is developmentally regulating. To test this, we immunostained both ankyrin G, a scaffolding protein of the axon initial segment (AIS) and Kv1.1 channels in thin slices of P11 and P25 rat dLGN. While at P25, Kv1.1 and ankyrin G were colocalized at the AIS, at P11, ankyrin G but not Kv1.1 was stained at the AIS (Fig. 5E). The quantification of the Kv1.1 immunostaining at the AIS indicates that it increases by a factor of 3 between P11 and P25 (Fig. 5E). As relay neurons represent ~85% of neurons in the dLGN, we conclude that the Kv1.1 staining obtained here most likely corresponds to relay neurons and not to GABAergic interneurons.

### A Hodgkin-Huxley–type model predicts experimental observations

**Fig. 6.**
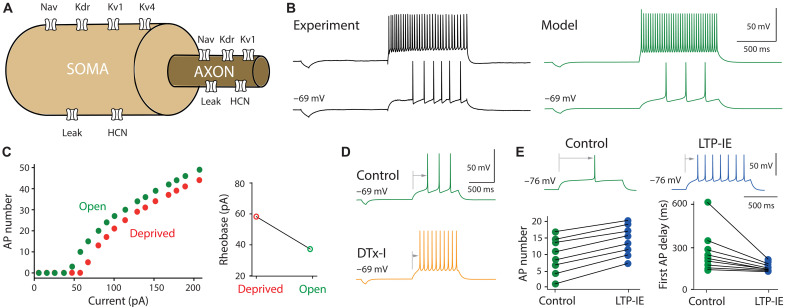
A two-compartment Hodgkin-Huxley model predicts experimental observations. (**A**) Model with simplified soma-axon geometry and localization of ion currents. (**B**) Example of experimental versus model voltage for two different current injections (42 pA, bottom; 180 pA, top). The membrane potential is set at −69 mV (+ 13 pA continuous current). (**C**) Left: Comparison of input-output curves from open (control, gKv1 = 41.7 nS in the soma and 23 nS in the axon) and deprived (increased gKv1 to 101.7 and 83 nS, respectively) model. Right: Comparison of the rheobase in open and deprived models. (**D**) Reproduction of the DTx-I effect by reducing gKv1 to 0 nS. Top: in control condition (green), the current step (here 47 pA) evokes three APs. Bottom: When gKv1 is set to 0 to simulate the DTx-I condition (orange trace), the same current pulse now evokes 10 APs. (**E**) Reduction of gKv1 by 50% qualitatively reproduces LTP-IE. The membrane potential is −76 mV (0 pA current). Top: Voltage traces obtained by injection of 45 pA before and after reducing gKv1 by 50%. Bottom: AP number and first spike delay in control (green) and LTP-IE (blue) models. The different data points have been obtained by varying the current intensity (from 45 to 59 pA).

To corroborate the involvement of Kv1 channels in LTP-IE expression in dLGN neurons, we built a two-compartment Hodgkin-Huxley model of thalamocortical cells, with a somato-dendritic compartment and an axonal compartment. The thalamocortical model is composed of various currents, partly based on a previous model (*24*), with the substantial addition of the Kv1 current (*25*, *26*). The localization of currents in compartments is based on experimental evidence in the literature (see Fig. 6A and Materials and Methods). To calibrate the model and to reproduce a set of features specific to the experimental voltage dynamics of the open eye, many parameters—including the maximum conductance and the half-activation of ionic currents—were estimated. Figure 6B shows an example of the model obtained against experimental data for two different depolarizing inputs (bottom: 40 pA; top: 180 pA). We can see that the model faithfully reproduces the dynamics of the data. Next, gKv1 was increased, and a decrease in the number of APs and an increase in rheobase were observed, as in the deprived eye (Fig. 6C). Then, to reproduce the application of DTx, gKv1 was reduced to 0, and an increase in the number of APs and a reduction in the delay of the first spike were observed (Fig. 6D). Last, to mimic LTP-IE, gKv1 was reduced by 50%. In this case, a mild elevation of intrinsic excitability and a reduction of first spike delay was obtained (Fig. 6E). Overall, the computational results corroborate the paramount involvement of Kv1 channels in activity-dependent plasticity of dLGN relay neurons.

### LTP-IE in MD rats

**Fig. 7.**
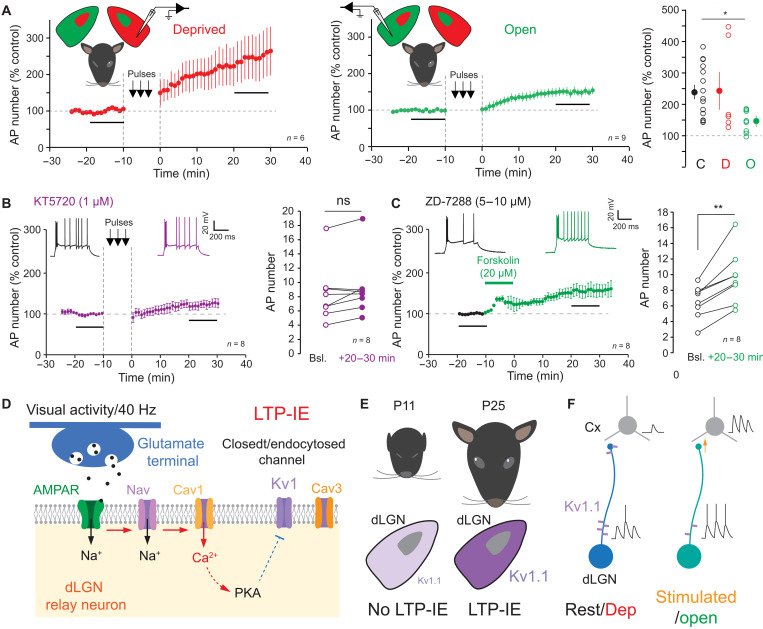
LTP-IE in deprived animals, requirement of PKA, and summary. (**A**) Reduced LTP-IE in dLGN neurons in amblyopia. Left: LTP-IE in dLGN neurons corresponding to the deprived eye. Middle: LTP-IE in dLGN neurons corresponding to the open eye. Right: Statistical comparison of LTP-IE in dLGN neurons from control (C), deprived (D), and open (O). Kruskal-Wallis, *P < 0.05. (**B**) Lack of LTP-IE in dLGN neurons recorded in the presence of the PKA inhibitor KT5720. Left: Time course. Right pooled data. Wilcoxon test. (**C**) PKA activator, forskolin, induces LTP-IE in dLGN neurons in the presence of the HCN channel blocker ZD-7288. Left: Time course with representative traces. Note the change in the latency of single action potentials after the initial burst. Right: Pooled data. Wilcoxon test, **P < 0.01. (**D** to **F**) Mechanisms of LTP-IE in dLGN neurons. (D) Glutamate release from retinal input activates AMPAR that depolarizes the membrane and activates Nav channels. L-type Cav1 channels activated by action potentials activates PKA. (E) At P11 (eyes closed), dLGN express little amounts of Kv1.1 channels, thus limiting LTP-IE expression. At P25, eyes are open and Kv1.1 are fully expressed, allowing the induction of LTP-IE. (F) Possible consequences of visual stimulation-induced loss-of-function of Kv1.1 channels on thalamo-cortical coupling. In both cases (rest and stimulated), similar inputs are delivered. Kv1.1 down-regulation increases spiking and reduces spike latency. In the cortex (Cx), the number of synaptic responses increases and their amplitude also because of reduction of Kv1 and spiking delay.

We next checked whether MD during 10 days interfered with LTP-IE. If vision enhances intrinsic neuronal excitability, an increase in LTP-IE would be expected in dLGN neurons corresponding to the deprived side, whereas a reduced LTP-IE is expected in dLGN neurons corresponding to the open eye. Compared to normally reared P19 animals (240 ± 22% of the control spike number, *n* = 15), LTP-IE was found to be significantly reduced in dLGN neurons activated by the open eye (149 ± 13% of the control spike number, *n* = 9; Kruskal-Wallis, *P* < 0.05) but not in neurons activated by the deprived eye in P22 to P23 animals (244 ± 60% of the control spike number, *n* = 6; Fig. 7A). We conclude that MD reduces the magnitude of LTP-IE in dLGN neurons activated by the open eye, suggesting that visual flow through the open eye prevents LTP-IE expression.

### LTP-IE in dLGN neurons requires PKA activity but not arachidonic acid

Kv1 channels are regulated by many molecules including lipids such as arachidonic acid (*27*, *28*). Inhibition of phospholipase A2 (i.e., the arachidonic acid synthesis enzyme) with AACOCF3 (20 μM) did not prevent the induction of LTP-IE (177 ± 18%, *n* = 6; fig. S8A), indicating that arachidonic acid is not involved in the down-regulation of Kv1 channels.

In visual cortical neurons, LTP-IE induced by a similar protocol (i.e., spiking activity at 40 Hz during 10 min) requires PKA activity (*15*). We therefore tested whether LTP-IE in dLGN neurons also involves PKA. Inhibition of PKA with KT5720 (1 μM) prevented LTP-IE (117 ± 10%, *n* = 8; AP number: 8 ± 1 before versus 9 ± 2 after, Wilcoxon, test, *P* > 0.05; Fig. 7B), indicating that activation of PKA is required for the induction of LTP-IE. Furthermore, application of the PKA activator forskolin (30 μM) during 10 min in the presence of the HCN channel blocker, ZD-7288 (1 μM), to prevent modulation of h-channels induced a mild LTP-IE (151 ± 11%, *n* = 8; Fig. 7C). AP number increased from 6.5 ± 0.7 to 9.6 ± 1.2, *n* = 8 after forskolin application (Wilcoxon test, *P* < 0.01; Fig. 7C). Here again, a hyperpolarization of the spike threshold (−36.1 ± 0.6 mV versus −37.1 ± 0.6 mV, *n* = 8; Wilcoxon test, *P* < 0.05; fig. S8B) and a reduction in the first spike latency after the burst (69 ± 6%, *n* = 8; mean latency: 223 ± 29 ms before and 152 ± 23 ms after stimulation, Wilcoxon test, *P* < 0.01; fig. S8C) were observed, suggesting that forskolin down-regulates Kv1 channels. Together, our results indicate that Ca^2+^ entry by L-type calcium channels activate PKA that in turn reduces Kv1 channel activity (Fig. 7D).

## DISCUSSION

We show here that MD reduces intrinsic excitability in dLGN relay cells of young rats, suggesting that visual activity enhances neuronal excitability in visual thalamic neurons. In addition, dLGN neurons display long-lasting enhancement of their intrinsic excitability upon stimulation of retinal inputs that elicit suprathreshold spiking activity. LTP-IE in dLGN relay neurons is also induced by spiking activity alone, indicating that synaptic activity does not play a critical role in its induction. Moreover, LTP-IE depends on a post-synaptic calcium influx that is not mediated by low-threshold T-type (Cav3) calcium channels but by high threshold L-type (Cav1) calcium channels. Furthermore, we show that LTP-IE in dLGN relay cells involves the down-regulation of Kv1 channels, as first spike latency is reduced and LTP-IE is blocked in the presence of DTx-I or DTx-K. LTP-IE is mediated by PKA activation but not production of arachidonic acid. LTP-IE could not be expressed before eye opening, indicating that it is developmentally regulated. Kv1.1 channel immunostaining was found at the AIS in P25 but not in P11 animals, suggesting that Kv1.1 channels may constitute the limiting factor for the expression of LTP-IE before eye opening (Fig. 7E). In MD animals, the amplitude of LTP-IE was found to be reduced on the open side but was comparable to the control on the deprived side, thus suggesting that visual flow through the open eye occludes further intrinsic potentiation. Our findings therefore constitute strong evidence for the presence of activity-dependent plasticity of intrinsic excitability in visual thalamic neurons both in vitro and in vivo.

### MD reduces intrinsic excitability in dLGN neurons

We show here that MD for 10 days reduces intrinsic neuronal excitability of dLGN neurons receiving the deprived eye inputs compared to dLGN neurons receiving the open eye inputs. A significant change in the rheobase was observed between the two populations of cells but no changes in input resistance nor in AP threshold were observed. This result strongly suggests that visual activity promotes neuronal excitability in dLGN relay neurons.

MD is responsible for a lack of visual acuity on the deprived eye, called the amblyopic eye. This loss of function is classically thought to result from receptive field size-dependent synaptic modifications. However, the reduction in responsiveness through the deprived eye observed after MD (*29*) may also result from changes in intrinsic neuronal excitability. A reduction in neuronal excitability has been reported following MD in L5 pyramidal neurons of the visual cortex (*18*). Our results therefore support the idea that inactive neurons become less excitable already at the thalamic stage. Furthermore, MD interacts with the expression of LTP-IE as LTP-IE is reduced in neurons activated by the open but not by the deprived eye.

### Induction mechanisms of intrinsic plasticity in dLGN relay cells

We show that LTP-IE in dLGN relay neurons was induced by synaptic stimulation at 40 Hz that produced spiking activity but not by subthreshold EPSPs, indicating that stimulation of post-synaptic receptors does not play a critical role in the induction of intrinsic plasticity, but that spiking activity is critical in LTP-IE induction. How spiking activity is naturally produced in dLGN neurons? In contrast to cortico-thalamic synapses that are positioned far in the dendritic arborization, retino-thalamic synapses are located near the cell body (*30*) and generate a large synaptic current able to trigger a post-synaptic AP (*31*). The requirement of spiking activity was further demonstrated by the lack of potentiation when no stimulation was delivered and by the fact that spiking activity induced by current steps (i.e., with no synaptic stimulation) induced LTP-IE of similar magnitude.

Spiking activity induces LTP-IE in dLGN neurons through a post-synaptic calcium influx mediated by L-type but not T-type calcium channels. T-type calcium channels have been shown to occupy a critical position in the induction of synaptic plasticity in subcortical neurons (*32*, *33*), but it does not play a critical role in LTP-IE induction in dLGN relay neurons. L-type calcium channels are involved in the induction of LTP and LTD in dLGN neurons (*34*) and in AIS plasticity (*35*). In addition, L-type calcium channels are known to play a critical role in retinogeniculate refinement as they contribute to the plateau potential activated by synaptic inputs (*36*).

### Stimulation parameters: Duration and frequency

We show that a minimal duration of 5 min is required to induce a significant increase in intrinsic excitability, as no significant enhancement of intrinsic excitability is observed with 2.5 min of 40-Hz stimulation. In addition, we show that no stimulation during 10 min induces no LTP-IE.

Firing frequency was also found to be critical as a stimulation at 40 Hz but not 10 Hz or 150 Hz induced a significant increase in intrinsic excitability in dLGN relay neurons. This may result from the lack of summation of calcium influx at 10 Hz but not at 40 Hz. During visual stimulation, dLGN relay neurons in rodents fire at mean frequencies ranging from ~5 to ~50 Hz (*37*, *38*). Thus, firing frequency at 40 Hz used to induce LTP-IE in dLGN relay neurons corresponds to a physiological firing frequency observed during visual stimulation. The 40-Hz frequency also corresponds to the binding and plasticity frequency in the visual cortex and to the intrinsic resonance frequency of thalamo-cortical neurons (*18*, *39*–*43*).

### Expression mechanisms of intrinsic plasticity in dLGN relay cells

Long-lasting intrinsic plasticity in cortical and hippocampal neurons involves a wide set of voltage-gated channels including HCN, SK, Kv1, and Kv7 channels (*13*, *14*, *23*, *44*–*46*). We show that Kv1 channels are involved in the expression of LTP-IE in dLGN neurons. First, consistent with a loss of function of the Kv1 channels (*23*), the latency of the first AP was reduced. Furthermore, the spike jitter was also reduced, and the spike threshold was elevated (*21*, *23*). Last, LTP-IE was occluded by DTx. Down-regulation of Kv1 channel activity does not involve the production of arachidonic acid. In contrast, PKA is involved in LTP-IE induction as its inhibition prevents LTP-IE induction, and its activation by forskolin enhances intrinsic excitability. The spike latency was also reduced by forskolin suggesting that PKA also targets Kv1 channels. The loss of function of Kv1 channels by PKA has been shown to be mediated by Kv1 channel endocytosis (*47*).

Whether these expression mechanisms identified in LTP-IE in vitro also occur during MD in vivo is still unclear. First, LTP-IE is monitored during a few tens of minutes in in vitro experiments without any sign of decrement, but it is not known whether this persists for hours or days. Second, the activity of thalamic neurons is controlled by many neuromodulators such as serotonin, acetylcholine, noradrenaline, and orexin (*48*–*53*) that may tune neuronal excitability during visual experience.

### Developmental regulation of LTP-IE

We report here that LTP-IE is developmentally regulated as no LTP-IE is observed before P17, corresponding to the onset of the critical period. However, it is observed up to P30 with no apparent decline. The fact that little or no LTP-IE is observed before P17 cannot be attributed to a possible depolarization block during the induction at 40 Hz because, even at P9, dLGN neurons exhibit a regular pattern without failures. A similar postnatal onset for plasticity (i.e., P17) has been recently reported for the plasticity of ocular dominance in the mouse dLGN (*54*). In addition, a shift in ocular dominance has been also reported in adult mouse dLGN (*11*). We show that Kv1.1 immunolabeling at the AIS increases by a factor of 3 from P11 to P25, suggesting that expression of Kv1.1 channels at the AIS may represent a limiting factor for the expression of LTP-IE.

### Reduction in first spike latency: A possible link between intrinsic and synaptic changes

One of the major functional consequences of LTP-IE in dLGN relay neurons is a reduction in the first spike latency, as a result of a reduction in Kv1 function. This effect is visible when the T-type calcium current is inactivated by pharmacological tools (TTA-P2) or by the depolarization caused by various neuromodulators (*55*). The major consequence of the Kv1-dependent latency shortening would be to promote the facilitation of the output synaptic message in the cortex (Fig. 7F) by a mechanism that depends on the minimization of axonal sodium channel inactivation (*56*). Although thalamo-cortical axons are >2 mm long in rodents (*57*), the presence of myelin prolongs their space constant up to 3 mm and thus makes possible the modulation of the output message (*58*). In addition, if the vision-induced reduction in Kv1 channels in dLGN neurons is not limited to the AIS but is also present at nerve terminals (*59*), an enhancement of the dLGN-cortical synaptic input is expected (Fig. 7E). Thus, intrinsic modifications at the stage of the dLGN may have synaptic consequences in the cortex.

### Implication for amblyopia

Amblyopia is characterized by a reduction of visual acuity through the eye that did not function properly during postnatal development. So far, this lack of visual function was thought to be located at the cortical stage. Our findings support the idea that functional changes already occur at the thalamic level as neuronal excitability is significantly elevated in neurons activated by the open eye compared to that of the deprived eye. The subthreshold ramp observed in neurons recorded within the deprived region suggests that firing is delayed for deprived neurons. This delayed firing will promote inactivation of sodium channels that would, in turn, weaken synaptic outputs at the cortical level (*56*).

A particularly interesting finding of our study is that LTP-IE is reduced in dLGN neurons activated by the open eye but not by the deprived eye. The magnitude of LTP-IE declined from 240% in control animals to 160% in neurons found in the contralateral projection region of the dLGN to the open eye, while it remained identical in the contralateral projection zone to the closed eye. The reduced LTP-IE in neurons activated by the open eye is consistent with an increased excitability caused by visual flow. An increase in LTP-IE has been observed in the visual cortex on the deprived side consistent with visual-dependent reduction of IE (*18*). However, in that study, the spared region had not been investigated.

Our findings have been obtained in the contralateral zone of the dLGN in which the synaptic drive from the ipsilateral eye is weak (*60*, *61*). Nevertheless, the purpose of our study was to compare neuronal excitability fed by one eye and the other in the context of amblyopia. Additional experiments will be required to further evaluate the changes in excitability in the ipsilateral region of the dLGN following MD and following dark rearing (*62*).

Compared to other studies in which the consequences of MD were tested after 2 to 3 days, the duration of eye closure is rather long in our study (i.e., 10 days). Further investigations will be necessary to check the effects of shorter durations of MD on intrinsic excitability of dLGN neurons.

## METHODS

### Monocular deprivation and identification of projection zones

All experiments were conducted according to the European and Institutional guidelines [council directive 86/609/EEC and French National Research Council and approved by the local health authority (Veterinary services, Préfecture des Bouches-du-Rhône, Marseille; APAFIS no. 7661-2016111714137296 v4)]. Young (P12 to P13) Long Evans rats of both sexes were anesthetized with isoflurane, and lids of the right eye were sutured for 10 days after adding eye drops containing a local anesthetic (tetracaine 1%). Sutures were checked each day, and if not intact, animals were not used.

P12 to P13 rats were deeply anesthetized with isoflurane, and the sclera and cornea were pierced with a syringe containing a 1% solution of cholera toxin B subunit (Invitrogen) conjugated to either Alexa Fluor 488 (green) or Alexa Fluor 555 (red) dissolved in distilled water to inject a different fluorescent marker in each eye (*63*). The animals were kept for 1 to 2 days after injection.

### Acute slices of rat dLGN

Thalamic slices (350 μm) were obtained from 9- to 30-day-old Long Evans rats of both sexes. Rats were deeply anesthetized with isoflurane and killed by decapitation. Slices were cut in an ice-cold solution containing 92 mM *n*-methyl-d-glutamine, 30 mM NaHCO_3_, 25 mM d-glucose, 10 mM MgCl_2_, 2.5 mM KCl, 0.5 mM CaCl_2_, 1.2 mM NaH_2_PO_4_, 20 mM Hepes, 5 mM sodium ascorbate, 2 mM thiourea, and 3 mM sodium pyruvate and bubbled with 95% O_2_ to 5% CO_2_ (pH 7.4). Slices recovered (20 to 30 min) in the *N*-methyl-d-glucamine solution before being transferred in a solution containing 125 mM NaCl, 26 mM NaHCO_3_, 2 mM CaCl_2_, 2.5 mM KCl, 2 mM MgCl_2_, 0.8 mM NaH_2_PO_4_, and 10 mM d-glucose and equilibrated with 95% O_2_ to 5% CO_2_. Each slice was transferred to a submerged chamber mounted on an upright microscope (Olympus, BX51 WI), and neurons were visualized using differential interference contrast infrared video microscopy.

### Electrophysiology

Whole-cell patch-clamp recordings were obtained from relay neurons in the contralateral projection zone of the dLGN. The external saline contained 125 mM NaCl, 26 mM NaHCO_3_, 3 mM CaCl_2_, 2.5 mM KCl, 2 mM MgCl_2_, 0.8 mM NaH_2_PO_4_, and 10 mM d-glucose and equilibrated with 95% O_2_ to 5% CO_2_. Synaptic inhibition was blocked with 100 μM picrotoxin. In experiments where LTP-IE was induced with current pulses, excitatory synaptic transmission was also blocked with 2 mM kynurenate. Patch pipettes (5 to 10 megohm) were pulled from borosilicate glass and filled with an intracellular solution containing 120 mM k-gluconate, 20 mM KCl, 10 mM Hepes, 0.5 mM EGTA, 2 mM MgCl_2_, 2 mM Na_2_ATP, and 0.3 mM NaGTP (pH 7.4). Recordings were performed with a MultiClamp-700B (Molecular Devices) at 30°C in a temperature-controlled recording chamber (Luigs & Neumann, Ratingen, Germany). The membrane potential was corrected for the liquid junction potential (−13 mV). In all recordings, access resistance was fully compensated using bridge balance and capacitance neutralization (>70%). dLGN neurons were recorded in current clamp, and input resistance was monitored throughout the duration of the experiments. Cells that display a variation >20% were discarded from final analysis. Voltage and current signals were low pass–filtered (10 kHz), and sequences of 2 s were acquired at 20 kHz with pClamp (Axon Instruments, Molecular Devices). Intrinsic excitability was tested with injection of current pulses (150 to 250 nA, 800 ms) to elicit three to four APs in control conditions at a frequency of 0.1 Hz. The amplitude of the current pulse was kept constant before and after induction of LTP-IE.

### Test of excitability after MD

The excitability of dLGN neurons after MD was tested at two different membrane potentials: −65 mV that approximately corresponds to the resting membrane potential of dLGN neurons and at −56 mV that corresponds to the transmission mode without bursting observed during wakefulness (*64*). Steps of depolarizing current with an increment of 10 pA were injected, and the number of AP was counted for each current value. The rheobase was determined as the smallest current that elicits at least an AP.

### Synaptic stimulation and induction of LTP-IE

Glass stimulating electrodes were filled with the extracellular medium. EPSPs were evoked in dLGN neurons with a pipette placed on the retino-thalamic endings of optic tract. Typically, EPSP amplitude reached its maximum value with stimulus intensity below 50 to 70 pA. The stimulus intensity was adjusted either to evoke an AP by each EPSP (intensity: 50 to 100 μA) or on the opposite, no AP (intensity: 20 to 40 μA). To mimic visual stimulation, trains of 15 synaptic stimulations at 40 Hz were applied during 10 min at a frequency of 0.1 Hz (i.e., 900 stimulations). In some cases, APs were evoked by short steps of current (2 to 5 ms, 1.0 to 2.5 nA) in the form of trains of 15 pulses delivered at a frequency of 40 Hz (or 10 Hz) during 10 minutes at a time interval of 10 s (i.e., 900 pulses). The amplitude of the current pulse was chosen to elicit a single AP by the current pulse (i.e., 15 APs per train).

### Voltage-clamp recording of Kv1 currents

To measure the D-type current mediated by Kv1 channels, before and after induction of LTP-IE with current pulses, dLGN neurons were recorded in voltage clamp in the presence of TTA-P2. Voltage commands from −80 to −50/−45 mV were applied and outward currents were obtained in control and after post-synaptic spiking for 10 min using a P/4 protocol to remove capacitive and resistive components. DTx-K was then bath-applied, and DTx-sensitive currents (i.e., Kv1 channel–mediated currents) were obtained in control and after current pulses by subtraction of the current traces obtained in DTx-K.

### Data analysis

Electrophysiological signals were analyzed with ClampFit (Axon Instruments, Molecular Devices). Spikes were counted using Igor Pro software (Wavemetrics). Input resistance was calculated from voltage response to small negative current pulses (typically, −20 pA, 250 ms). LTP-IE was measured over a period of 10 min, 20 min after the beginning of the post-stimulation period.

### Drugs

BAPTA was added to the intracellular solution and was obtained from Tocris Bioscience. All other chemicals were bath applied. Picrotoxin and arachidonyl trifluoromethyl ketone (AACOCF3) were purchased from Abcam; DTx-I, DTx-k, and TTA-P2 from Alomone and (3*R*, 4a*R*, 5*S*, 6*S*, 6a*S*, 10*S*, 10a*R*, 10b*S*)-5-(Acetyloxy)-3-ethenyldodecahydro-6,10,10b-trihydroxy-3,4a,7,7,10a-pentamethyl-1*H*-naphtho[2,1-*b*] pyran-1-one (forskolin), 9*R*,10*S*,12*S*)-2,3,9,10,11,12-hexahydro-10-hydroxy-9-methyl-1-oxo-9,12-epoxy-1*H*-diindolo[1,2,3-*fg*:3′,2′,1′-*kl*] pyrrolo[3,4-*i*] [1,6] benzodiazocine-10-carboxylic acid, hexyl ester (KT5720); and nitrendipine were purchased from Tocris Bioscience.

### Immunostaining

Postnatal P11 and P25 wild-type Long Evans rats were deeply anesthetized and perfused with ice-cold 4% paraformaldehyde diluted in 20 mM glucose in 0.1 M phosphate buffer (PB; pH 7.4). Brains were removed from the skull and post-fixed overnight (o/n) at 4°C in the same fixative solution. Sections (70-μm thick) were cut using Leica VT1200S vibratome and then processed for immunohistochemical detection. Brain slices were washed in Dulbecco’s phosphate-buffered saline (Gibco, #14190-094) and incubated 30 min in 10 mM sodium citrate (pH 8.5) at 80°C for antigen retrieval. Then, there washed with 0.1 M PB (pH 7.4). Immunodetection was done in free-floating sections. Brain slices were treated with 50 mM NH_4_Cl for 30 min and incubated in blocking buffers to diminish nonspecific binding using a first-step PB with 1% (w/v) bovine serum albumin (BSA) (Sigma-Aldrich, #A3059), 0.3% (v/v) Triton X-100 (Merk, #648463) for 30 min and then PB with 5% (w/v) BSA, 0.3% (v/v) Triton X-100 for 2 hours. Slices were incubated overnight at 4°C with primary antibodies, mouse anti-Kv1.1 clone K36/15 IgG2b (5 μg/ml) from Sigma-Aldrich, and rabbit anti-ankyrinG (2.5 μg/ml) from Synaptic System (#386003), both diluted in incubation buffer [1% (w/v) BSA and 0.3% (v/v) Triton X-100 in PB]. After extensive washing in incubation buffer, the secondary antibodies, Alexa Fluor 488 donkey anti-mouse (2 μg/ml; #715-546-151) and Alexa Fluor 594 donkey anti rabbit (2 μg/ml; #711-586-152) from Jackson Immunoresearch were incubated for 2 hours at room temperature and then washed. After staining, the coverslips were mounted with VECTASHIELD Vibrance (Vector Laboratories, H-1700-10).

### Confocal fluorescence microscopy and image analysis

Images were acquired on a confocal laser scanning microscope (LSM 780 Zeiss) using the same settings to compare intensities between experimental conditions P11 and P25 ages. All images were acquired using a Plan-Apo 63× /1.4 oil-immersion objective lens. All confocal images were acquired at 0.38-μm *z* axis steps and with a 1024 × 1024 pixel resolution. Photons emitted from the two dyes were detected sequentially with one photomultiplier tube for each dye to minimize cross-talk. Images were prepared using Adobe Photoshop.

Analysis was performed using the ImageJ software (National Institutes of Health). Images stacks were converted into single maximum intensity *z* axis projections. AIS was identified by AnkyrinG staining and was selected with threshold determination of fluorescent labeling area (red stain). After subtraction of the background, average gray value was measured on Kv1.1 staining within the selection (the sum of the gray values of all the pixels in the selection divided by the number of pixels). All statistical analyses were carried out in SigmaPlot.

### Statistics

Pooled data are presented as means ± SEM. Statistical analysis was performed using Wilcoxon test, Mann-Whitney *U* test, or Kruskal-Wallis test.

### Hodgkin-Huxley type model

A compartmental Hodgkin-Huxley model was implemented in Brian2 (*65*). The currents that compose the model (see Supplementary Materials) are partly based on those of McCormick and Huguenard (*25*, *26*), with the substantial addition of the Kv1 current. The geometry consisted of a somato-dendritic compartment and an axonal compartment. The localization of ionic currents in these compartments, summarized in Fig. 6A, was based on a series of experimental evidence. In particular, there is strong evidence for the localization of the A-type current in the somato-dendritic compartment of many different cell types (*66*, *67*). The Kv1 current is expressed both in the AIS and in the somato-dendritic compartment, as previously shown (*68*). A sodium current for AP generation and a delayed-rectifier potassium current were also included in both compartments, along with a leakage current and an HCN current, as these are known to shape the resting membrane potential and input resistance of thalamocortical relay cells (*24*, *69*). Each current was specified as a set of differential equations following the Hodgkin-Huxley formalism. To calibrate the model, parameter estimation was carried out using the brian2modelfitting package (*70*). More specifically, the maximal conductance and the half-activation of ionic currents were estimated to fit specific features of the voltage dynamics (*71*), such as the AP number and amplitude, the time to first spike, the input resistance, and the resting potential (Supplementary Materials). The optimization method used in this paper was the differential evolution algorithm (*72*) as it has been shown not only to be an effective method (*73*, *74*) but also superior to other optimization methods such as genetic algorithms, simulated annealing and particle swarm optimization algorithm in terms of convergence speed, simulation time, and minimization of the cost function (*75*).

## Supplementary Materials

### This PDF file includes:

Supplementary Text Table S1 Figs. S1 to S8 References
