# Supplementary material for: Visual activity enhances neuronal excitability in thalamic relay neurons
Source: Sci Adv. 2025 Jan 22;11(4):eadp4627. doi: 10.1126/sciadv.adp4627 (PMC11753433; doi:10.1126/sciadv.adp4627)
Supplement: Supplementary file 1 — Supplementary Text Table S1 Figs. S1 to S8 References [file sciadv.adp4627_sm.pdf]

Science Advances
AAAS

Supplementary Materials for

Visual activity enhances neuronal excitability in thalamic relay neurons

Maël Duménieu *et al.*

Corresponding author: Dominique Debanne, dominique.debanne@inserm.fr;
Michaël Russier, michael.russier@univ-amu.fr

Sci. Adv. **11**, eadp4627 (2025)
DOI: 10.1126/sciadv.adp4627

This PDF file includes:

Supplementary Text
Table S1
Figs. S1 to S8
References

Model

Our model is partly based on the one from (*76*). It takes the form

$$C\frac{dV}{dt} = -I_{Na,t} - I_{Kv1} - I_A - I_{Kdr} - I_L - I_H + I$$

where V denotes the membrane potential; C the membrane capacitance; $I_{Na,t}$ a transient sodium current; I_{Kv1}, I_A and I_{Kdr} three potassium currents (a Kv1 current, an A-type current, and a delayed rectifier current, respectively); I_H a hyperpolarization-activated cyclic nucleotide-gated channel; I_L a leak current; and I an artificial injection current. Each of these currents follow the classical Hodgkin-Huxley formalism:

$$I_{ion} = g_{ion} m_{ion}^a h_{ion}^b (V - E_{ion})$$

where m (resp. h) denotes the probability for an activation (resp. inactivation) gate to be in the open state; a and b are the number of activation and inactivation gates, respectively; g_{ion} is the maximal conductance associated with ion; and E_{ion} is the reversal potential.

We now further describe the modelling of each of these currents. All the estimated parameter values are displayed in Table 1, while other parameter values come from previous models (*76*) and are indicated in the text.

Potassium channels

Three potassium currents were taken into account: an A-type current (I_A) that is the most prominent in thalamocortical neurons (*77*), a Kv1 current (I_{Kv1}), also known as D-current, and a delayed rectifier current (I_{Kdr}). The A-type current is modeled as in (*77*) as the sum of two currents (I_{A1} and I_{A2}) with comparable activation kinetics and steady-state inactivation, but with different steady-state activation:

$$I_{A1} = 0.6 g_A m_{A1}^4 h_{A1} (V - E_K)$$

$$I_{A2} = 0.4 g_A m_{A1}^4 h_{A2} (V - E_K)$$

with $E_K = -90$ mV, and m_{A1} and m_{A2} (resp. h_{A1} and h_{A2}) the activation (resp. inactivation) gating variables which take the form

$$\tau_x \frac{dx}{dt} = \left(1 + exp\left(\left(V_{1/2}^x - V\right)/k_x\right)\right)^{-1} - x, \qquad x \in \{m_{A1}, m_{A2}, h_{A1}, h_{A2}\},$$

with $V_{1/2}^{m_{A1}} = -60$ mV, $k_{m_{A1}} = 8.5$ mV, $V_{1/2}^{m_{A2}} = -36$ mV, $k_{m_{A2}} = 20$ mV, $k_{h_{A1}} = k_{h_{A2}} = 6$ mV, and $V_{1/2}^{h_{A1}} = V_{1/2}^{h_{A2}}$ as free parameters to be estimated.

Time constants reads as

$$\tau_{m_{A1}} = \tau_{m_{A2}} = \left(0.37 + exp\left((V + 35.8)/19.7\right) + exp((V + 79.7)/-12.7\right)^{-1}$$

$$\tau_{h_{A1}} = \left(exp\left((V + 46.05)/5\right) + exp((V + 238.4)/-37.45\right)^{-1} \, if\ V < -63\ and\ \tau_{h_{A1}} = 19\ if\ V\ \geq -63$$

$$\tau_{h_{A2}} = \left(exp\left((V + 46.05)/5\right) + exp((V + 238.4)/-37.45\right)^{-1} \, if\ V < -73\ and\ \tau_{h_{A2}} = 60\ if\ V\ \geq -73$$

with time in ms and voltage in mV.

The Kv1 current is modelled as in (*26*) based on experimental data from (*25*), with an additional inactivation variable:

$$I_{Kv1} = g_{Kv1} m_{Kv1}^8 \left(0.35 h_{Kv1}^f + 0.65 h_{Kv1}^s\right)(V - E_K)$$

with two components of the inactivation variable, a fast one (h_{Kv1}^f) and a slow one (h_{Kv1}^s), which are modelled as follows:

$$\tau_x \frac{dx}{dt} = \left(1 + exp\left(\left(V_{1/2}^x - V\right)/k_x\right)\right)^{-1} - x, \qquad x \in \{h_{Kv1}^f, h_{Kv1}^s\},$$

with $k_{h_{Kv1}^f} = k_{h_{Kv1}^s} = -12.1$ mV, and $V_{1/2}^x$ and τ_x free parameters to be estimated. The activation variable m_{Kv1} is governed by a standard kinetic equation

$$\frac{dm_{Kv1}}{dt} = \alpha_{m_{Kv1}}(1 - m_{Kv1}) - \beta_{m_{Kv1}} m_{Kv1}$$

where $\alpha_{m_{Kv1}}$ and $\beta_{m_{Kv1}}$ are opening and closing rates, respectively, which take the form:

$$\alpha_{m_{Kv1}}(V) = \frac{1}{2k_{m_{Kv1}}\tau^*_{m_{Kv1}}} \frac{V - V_{1/2}^{m_{Kv1}}}{1 - exp\left(-\left(V - V_{1/2}^{m_{Kv1}}\right)/k_{m_{Kv1}}\right)}$$

$$\beta_{m_{Kv1}(V)} = -\frac{1}{2k_{m_{Kv1}}\tau^*_{m_{Kv1}}} \frac{V - V_{1/2}^{m_{Kv1}}}{1 - exp\left(\left(V - V_{1/2}^{m_{Kv1}}\right)/k_{m_{Kv1}}\right)}$$

with $k_{m_{Kv1}} = 20$ mV, and $V_{1/2}^{m_{Kv1}}$ and $\tau^*_{m_{Kv1}}$ free parameters to be estimated.

The delayed rectifier potassium channel takes the form

$$I_{Kdr} = g_{Kdr} m_{Kdr}^4 (V - E_K).$$

where m_{Kdr} the activation variable of the delayed rectifier potassium channel, whose dynamics is governed by the equation

$$\tau_{m_{Kdr}} \frac{dm_{Kdr}}{dt} = \left(1 + exp\left(\left(V_{1/2}^{m_{Kdr}} - V\right)/k_{m_{Kdr}}\right)\right)^{-1} - m_{Kdr}$$

with $\tau_{m_{Kdr}} = 1$ ms, $k_{m_{Kdr}} = 15$ mV and $V_{1/2}^{m_{Kdr}}$ a free parameter to be estimated.

Sodium channel
The transient sodium channel is

$$I_{Na} = g_{Na} m_{Na}^3 h_{Na} (V - E_{Na})$$

with $E_{Na} = 58$ mV, and m_{Na} and h_{Na} the activation and inactivation gating variables, respectively, which take the form

$$\tau_x \frac{dx}{dt} = \left(1 + exp\left(\left(V_{1/2}^x - V\right)/k_x\right)\right)^{-1} - x, \qquad x \in \{m_{Na}, h_{Na}\},$$

with τ_x and $V_{1/2}^x$ free parameters to be estimated.

Channels involved in the resting membrane potential setting

Two currents are involved in the resting membrane potential setting: a leak current and a HCN current. The leak current takes the form

$$I_L = g_L (V - E_L)$$

with $g_L = 0.2021$ S/m² and $E_L = -78$ mV, while the HCN current is described as in (*76*):

$$I_{hcn} = g_{hcn} h_{hcn} (V - E_{hcn})$$

with $g_{hcn} = 0.02$ mS/cm² and $E_{hcn} = -43$ mV, while the variable h_{hcn} reads as:

$$\tau_{\text{hcn}} \frac{dh_{hcn}}{dt} = \left(1 + exp\left(\left(V_{1/2}^{h_{hcn}} - V\right) / k_{h_{hcn}}\right)\right)^{-1} - h_{hcn},$$

with $k_{h_{hcn}} = 5.5$ mV, $V_{1/2}^{h_{hcn}}$ a free parameter to be estimated, and

$$\tau_{hcn}(V) = \left(\exp(-14.59 - 0.086\text{V}) + \exp(-1.87 + 0.0701\text{V})\right)^{-1}$$

**Passive properties**	$\boldsymbol{C_{soma}}$	1 µF/cm²
	C_{axon}	1 µF/cm²
	E_L	-78 mV
	g_L	0.2021 S/m²
	$Area_{somato-dendritic}$	10500 µm²
	$Area_{axon}$	290 µm²
	R_a	5.97 MΩ
**A-type current**	$g_{A,somato-dendritic}$	86.3 nS
	$V_{1/2}^{h_{A1}} = V_{1/2}^{h_{A2}}$	-32.3 mV
**Kv1 current**	$g_{Kv1,somato-dendritic}$	41.7 nS
	$g_{Kv1,axon}$	23 nS
	$\tau^*_{m_{Kv1}}$	3.9 ms
	$\tau_{\text{h}^{\text{f}}_{\text{Kv1}}}$	211.6 mS
	$\tau_{\text{h}^{s}_{\text{Kv1}}}$	4.57 s
	$V_{1/2}^{m_{Kv1}}$	-55.7 mV
	$V_{1/2}^{h_{Kv1}}$	-55.4 mV
**Kdr current**	$g_{Kdr,soma}$	77.7 nS
	$g_{Kdr,axon}$	76.7 nS
	$V_{1/2}^{m_{Kdr}}$	-26.27 mV
**Na channel**	$g_{Na,soma}$	1360 nS
	$g_{Na,axon}$	7096 nS
	$\tau_{m_{Na}}$	0.39 ms
	$\tau_{h_{Na}}$	2 ms
	$V_{1/2,soma}^{m_{Na}}$	-33.4 mV
	$V_{1/2,soma}^{h_{Na}}$	-61.9 mV
	$V_{1/2,axon}^{m_{Na}}$	$V_{1/2,soma}^{m_{Na}} - 5\,mV$
	$V_{1/2,axon}^{h_{Na}}$	$V_{1/2,soma}^{h_{Na}} - 5\,mV$
**HCN current**	$V_{1/2}^{h_{hcn}}$	-90.7 mV

Table S1. Estimated parameter values of the model

All the code is available at https://zenodo.org/records/10663967

Parameter estimation procedure

The estimated parameters obtained in Table S1 were obtained using the *brian2modelfitting* package in order to fit specific features of the voltage dynamics (*71*). These features included the AP number and amplitude, the time to first spike, the input resistance, and the resting potential. The error associated with each feature is quantified by the absolute difference between its experimental value (f^{exp}) and the model value (f^{est}). In other words, for each feature i, we consider the error $F_i := \left|f_i^{exp} - f_i^{est}\right|$. Therefore, the quality of a solution θ is the sum of the errors associated with each of these features, namely

$$F(\theta) = \sum_i \omega_i F_i$$

where ω_i is the relative weight of the feature i. To find an optimal individual θ that minimizes the cost function F, we used the differential evolution algorithm (*72*) as it has not only been shown to be an effective method (*73*, *74*), but also superior to other optimization methods such as genetic algorithms, simulated annealing and particle swarm optimization algorithm in terms of convergence speed, simulation time, and minimization of the cost function (*75*). The population size of the algorithm was set to 40 individuals, the number of generations to 400, and the crossover and mutation rates to 0.8.

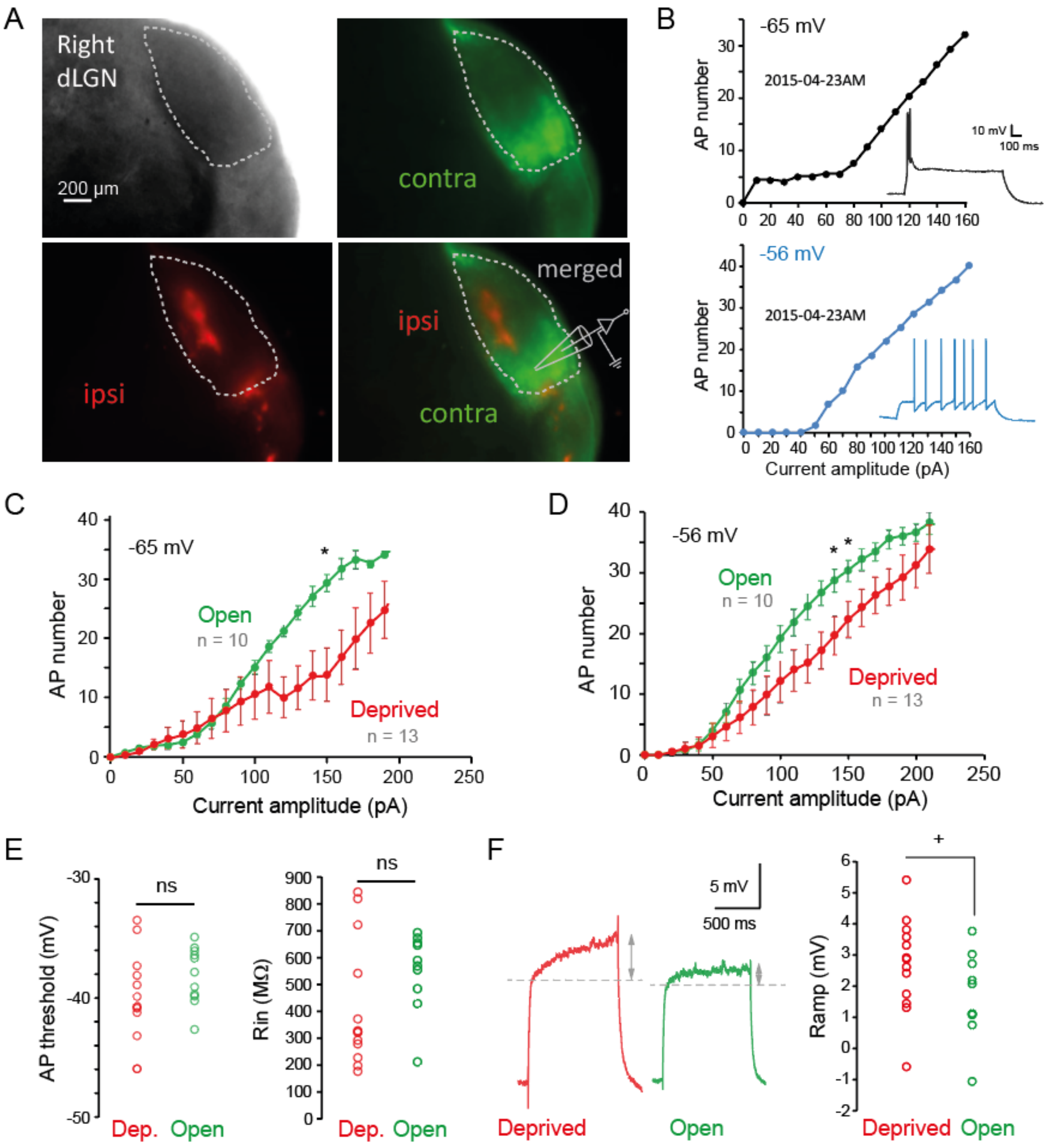

Fig. S1. Definition of the retinal projections in the rat dLGN and changes in excitability.
A. Labelling of the contralateral and ipsilateral retinal projections. B. Inactivation of the T-type calcium current reveals a delayed firing. At resting membrane potential (-65 mV, top), the rheobase is difficult to define because of the burst mediated by T-type calcium channels (see trace in response to 60 pA). However, when the same neuron is depolarized to -56 mV (bottom), the T-type current is inactivated (see trace in response to 60 pA) and the rheobase can be properly defined. C. Comparison of the input-output curves of open and deprived dLGN neurons measured at -65 mV. *, Mann-Whitney test, p <0.05. D. Pooled input-output curves for deprived and open dLGN neurons recorded at -56 mV. *, Mann-Whitney test, p <0.05. E. Comparison of the input resistance (Rin, left) and of the AP threshold (right). F. Comparison of subthreshold voltage ramps in neurons activated by the deprived and open eyes. The voltage ramp was found to be slightly larger in deprived neurons compared to open ones. Left, representative traces. Right, group data. Mann Whitney test, +, p<0.08.

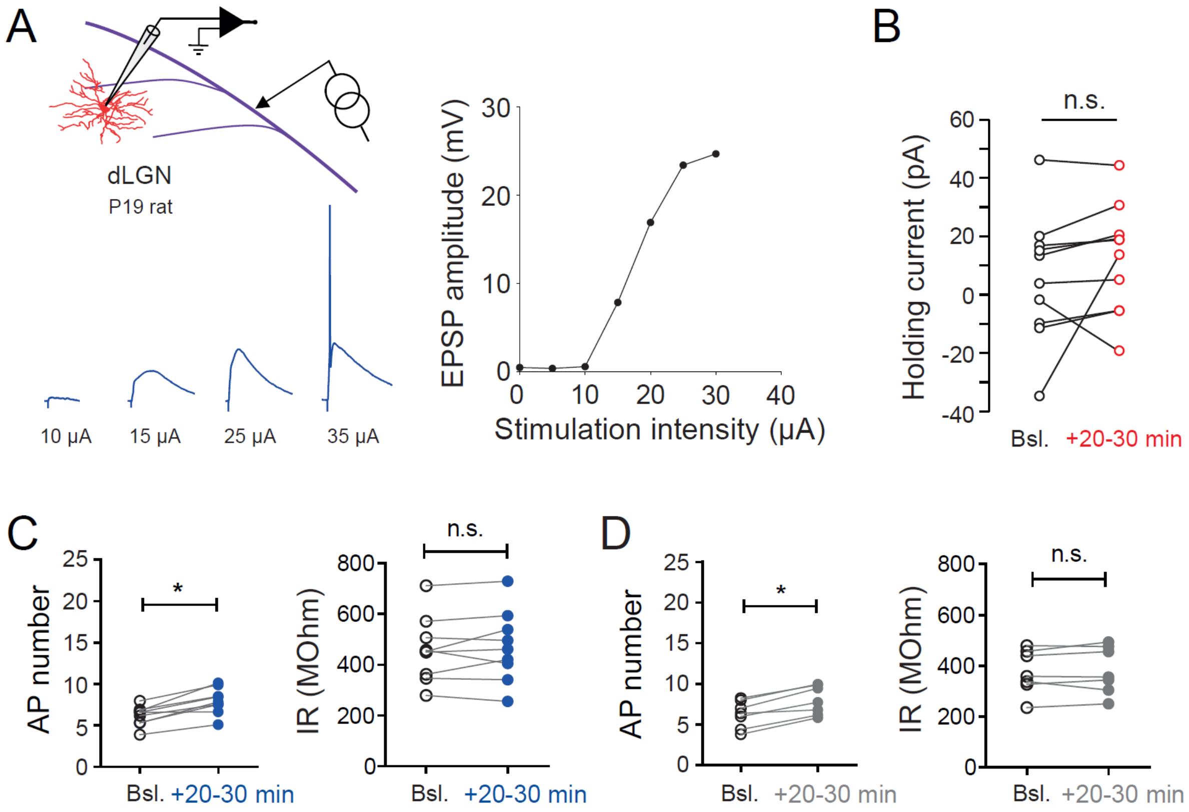

Fig. S2. Synaptic responses, AP number and input resistance.
A. Top left, recording and stimulation configuration. Bottom left, synaptic responses at different intensities. Note that at 35 µA, an AP is systematically evoked. Right, plot of the EPSP amplitude as a function of stimulus intensity. B. Holding current is not significantly changed. C & D. AP number and input resistance (IR) for subthreshold EPSPs (C) and no stimulation (D). Wilcoxon test, n.s., not significant, *, $p<0.05$.

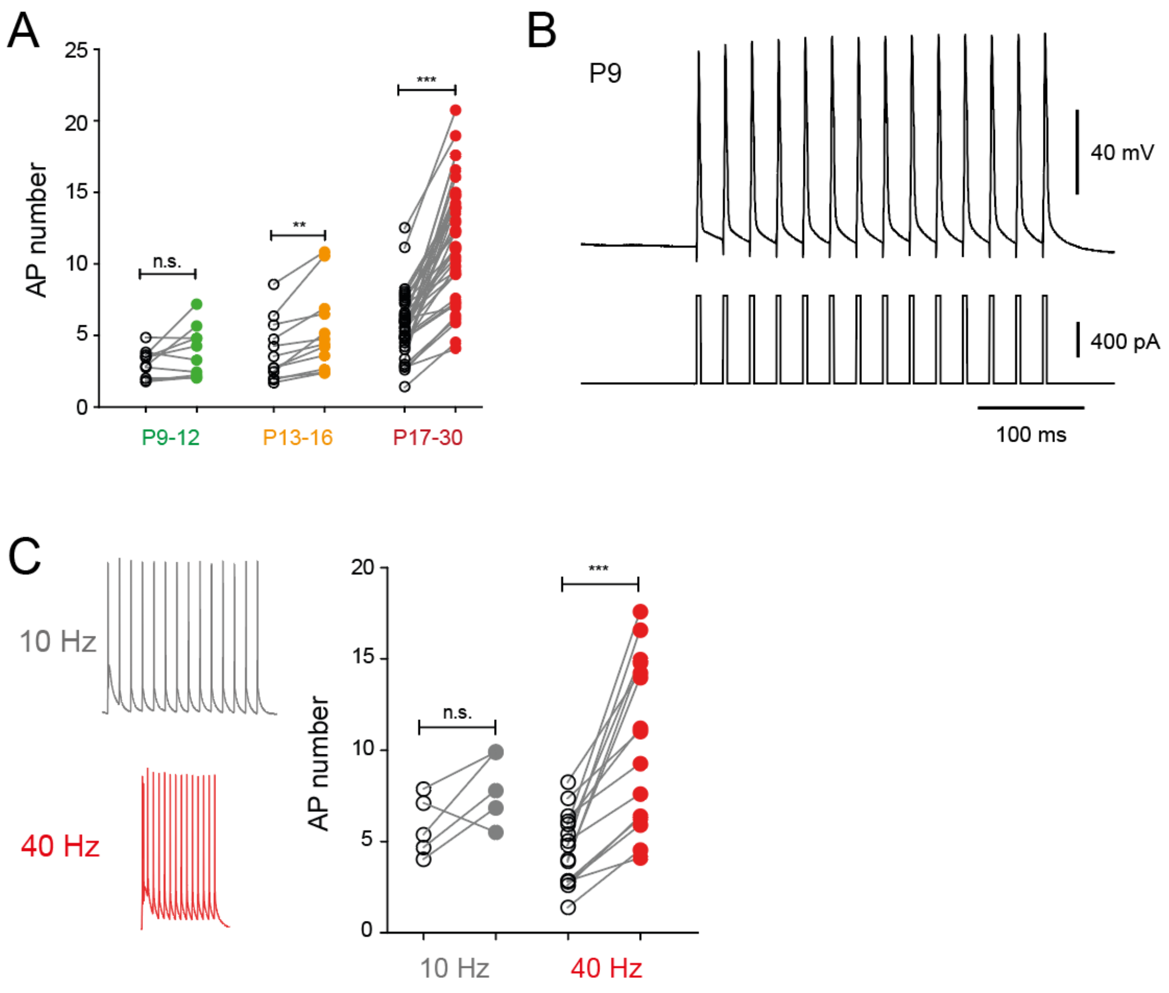

Fig. S3. Age- and frequency-dependence of LTP-IE in dLGN neurons.
A. Changes in spike number in each age category. Wilcoxon test, ns, not significant; **, p<0.01; ***, p<0.001. B. Firing profile during the induction in a P9 dLGN neuron. C. Frequency-dependence of LTPI-IE. Left, trace examples at 10 Hz (grey) and 40 Hz (red). Right, AP changes. Wilcoxon, ns, not significant; ***, p<0.001.

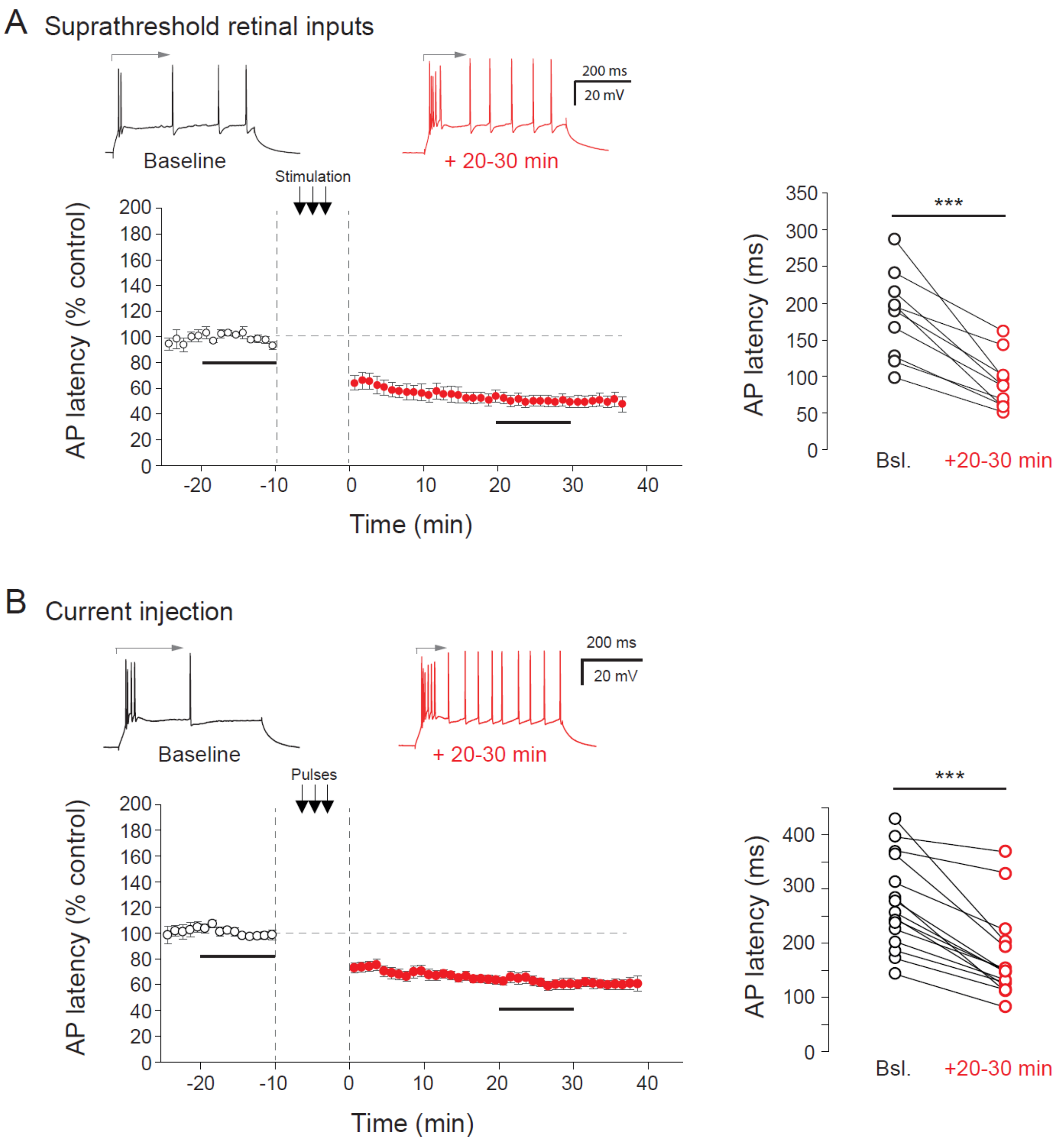

Fig. S4. Reduction of the AP latency following induction of LTP-IE.
A. Reduction of the AP latency of the first spike after the burst (indicated by an arrow) after induction of LTP-IE by stimulation of suprathreshold retinal inputs at 40 Hz. Left, time course. Right, group data. Wilcoxon test, ***, $p<0.001$. B. Reduction of the AP latency of the first spike after the burst (indicated by an arrow) after induction of LTP-IE by current pulse injection at 40 Hz. Left, time course. Right, group data. Wilcoxon test, ***, $p<0.001$.

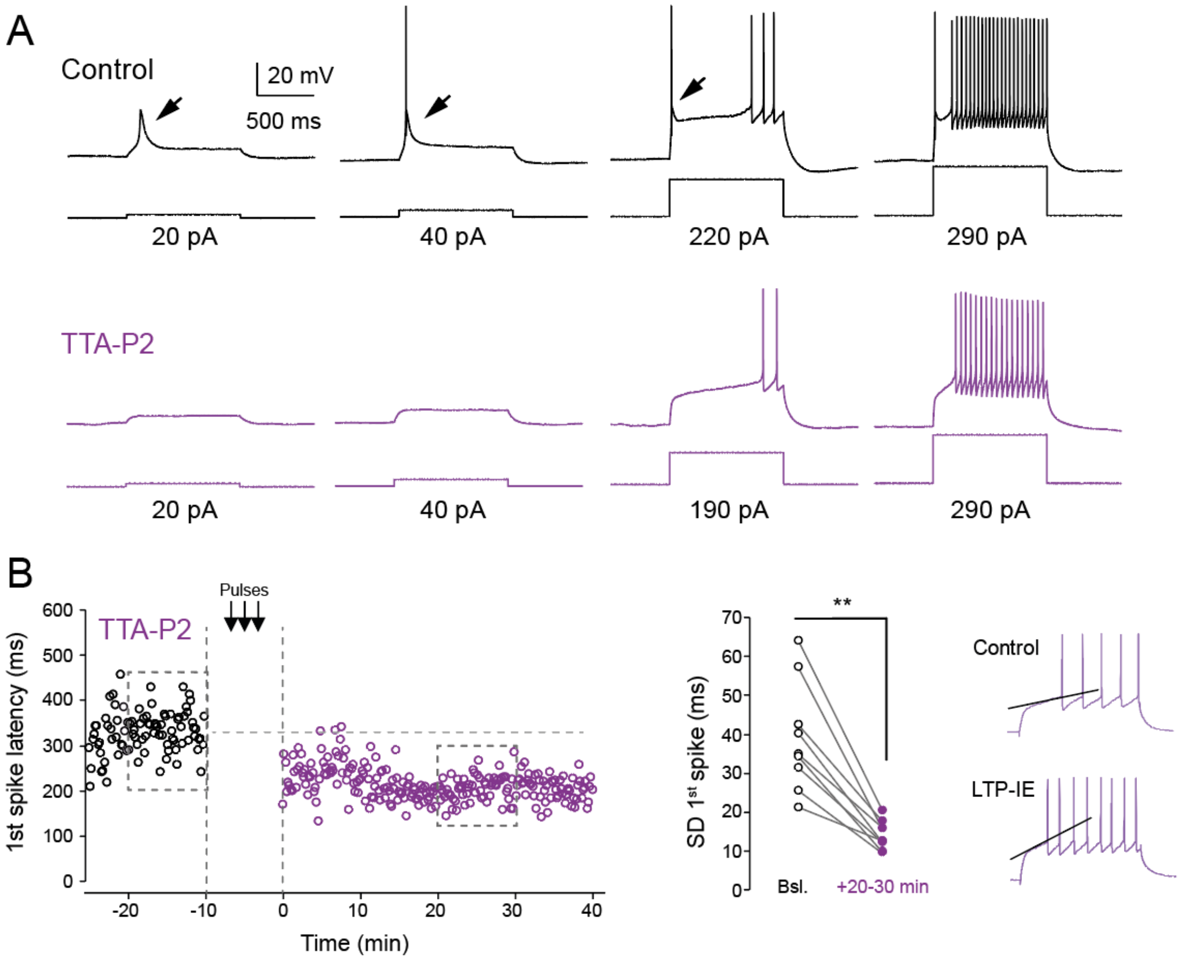

Fig. S5. Suppression of T-type potential by TTA-P2 and reduced spike jitter following LTP-IE induction. A. TTA-P2 blocks the T-type calcium potential in relay dLGN neurons. Top, in control a T-type calcium potential is observed at the onset of the depolarizing pulse (black arrows). Bottom, TTA-P2 totally suppress the T-type potential. B. Left, time-course of the first spike jitter in a neuron recorded in the presence of TTA-P2. Middle, group data. **, Wilcoxon test, $p<0.01$. SD, standard deviation. Right, slope changes before and after induction of LTP-IE.

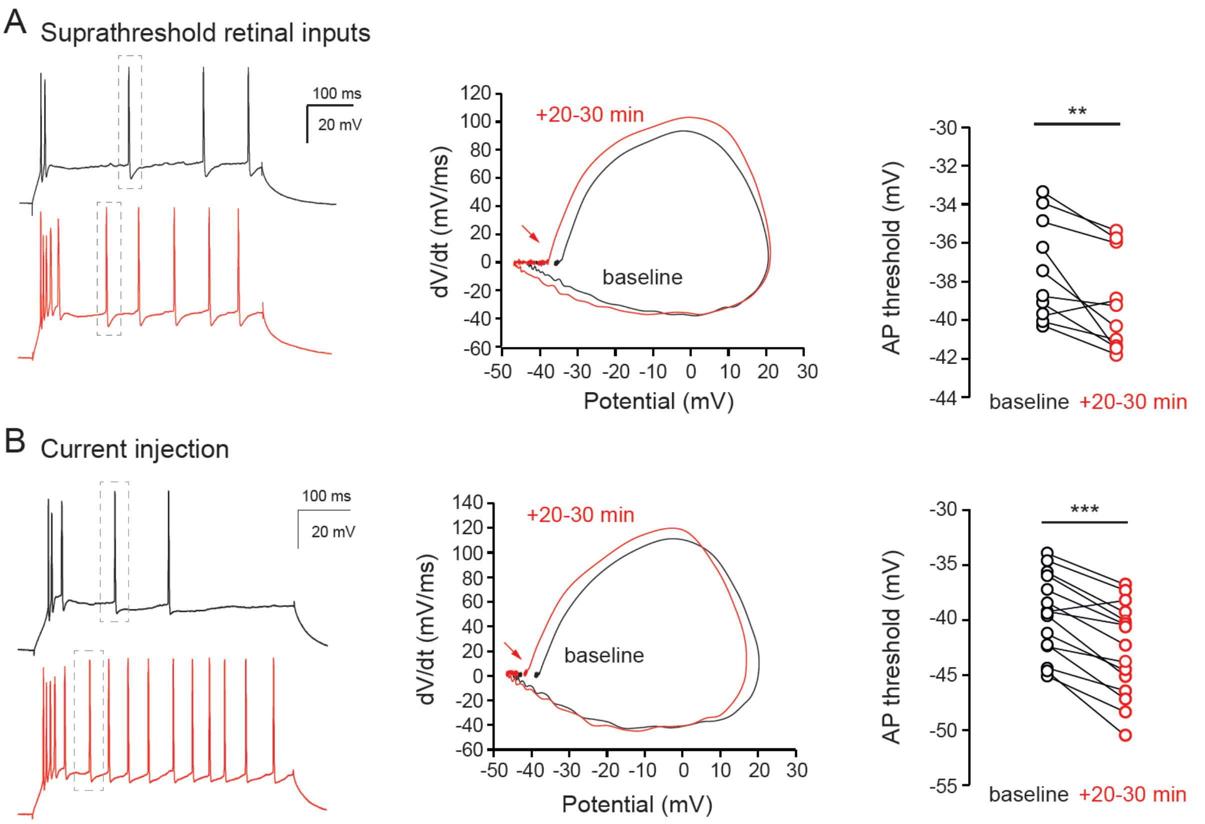

Fig. S6. Hyperpolarization of the AP threshold after induction of LTP-IE in dLGN neurons.
A & B. Hyperpolarization of the AP threshold after induction of LTP-IE with stimulation of suprathreshold retinal inputs (A) and by current injection (B). Left, representative traces. Middle, phase plots. Right, group data. Wilcoxon test, ** $p<0.01$; ***, $p<0.001$.

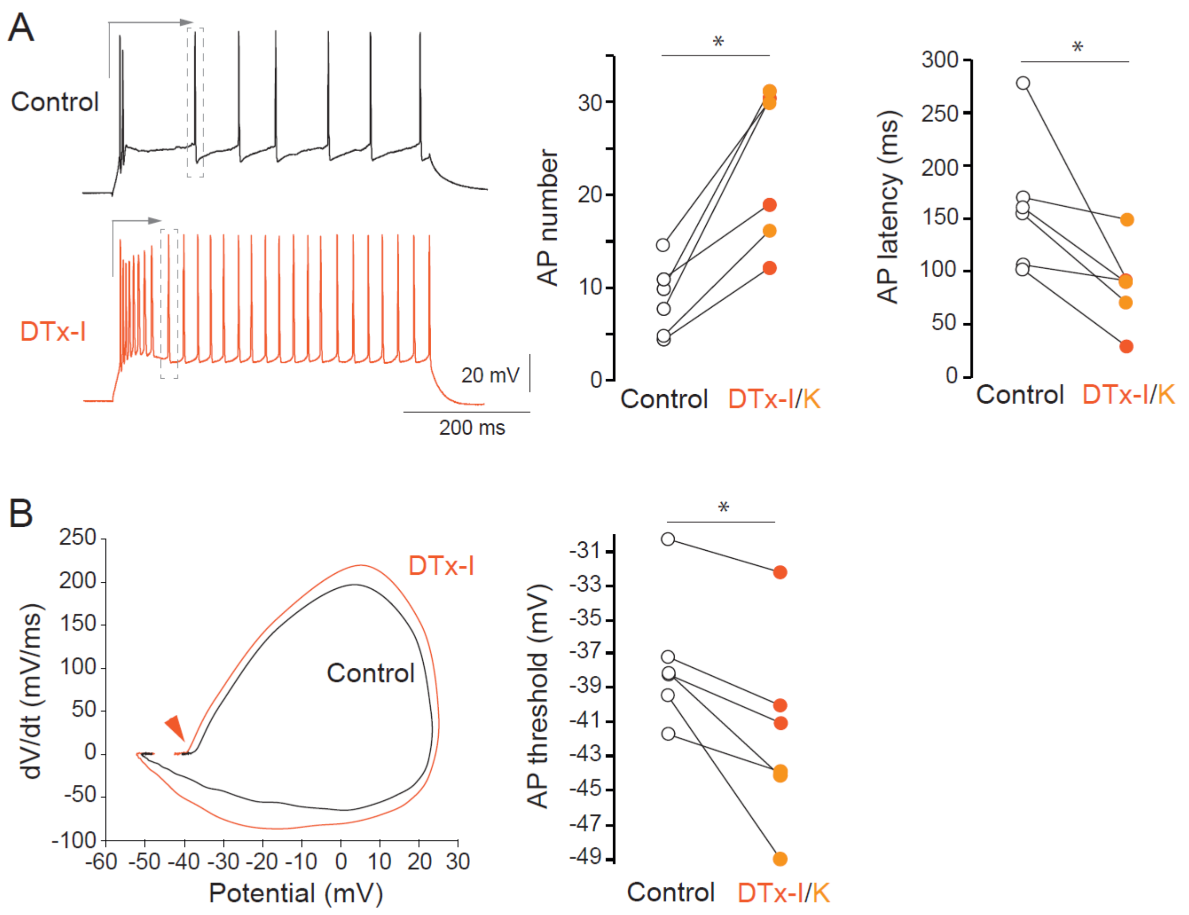

Fig. S7. Action of DTx on firing properties of dLGN neurons.
A. DTx increases evoked firing and reduces the delay of the first spike after the burst. Left, traces in control (black) and in DTx-I (orange). Right, pooled data for AP number and AP latency. Orange data points correspond to DTx-I and light-orange ones to DTx-K. B. DTx hyperpolarizes the AP threshold. Left, phase plots in control and DTx-I. Right, pooled data. Orange data points correspond to DTx-I and light-orange ones to DTx-K. Wilcoxon test, * $p<0.05$.

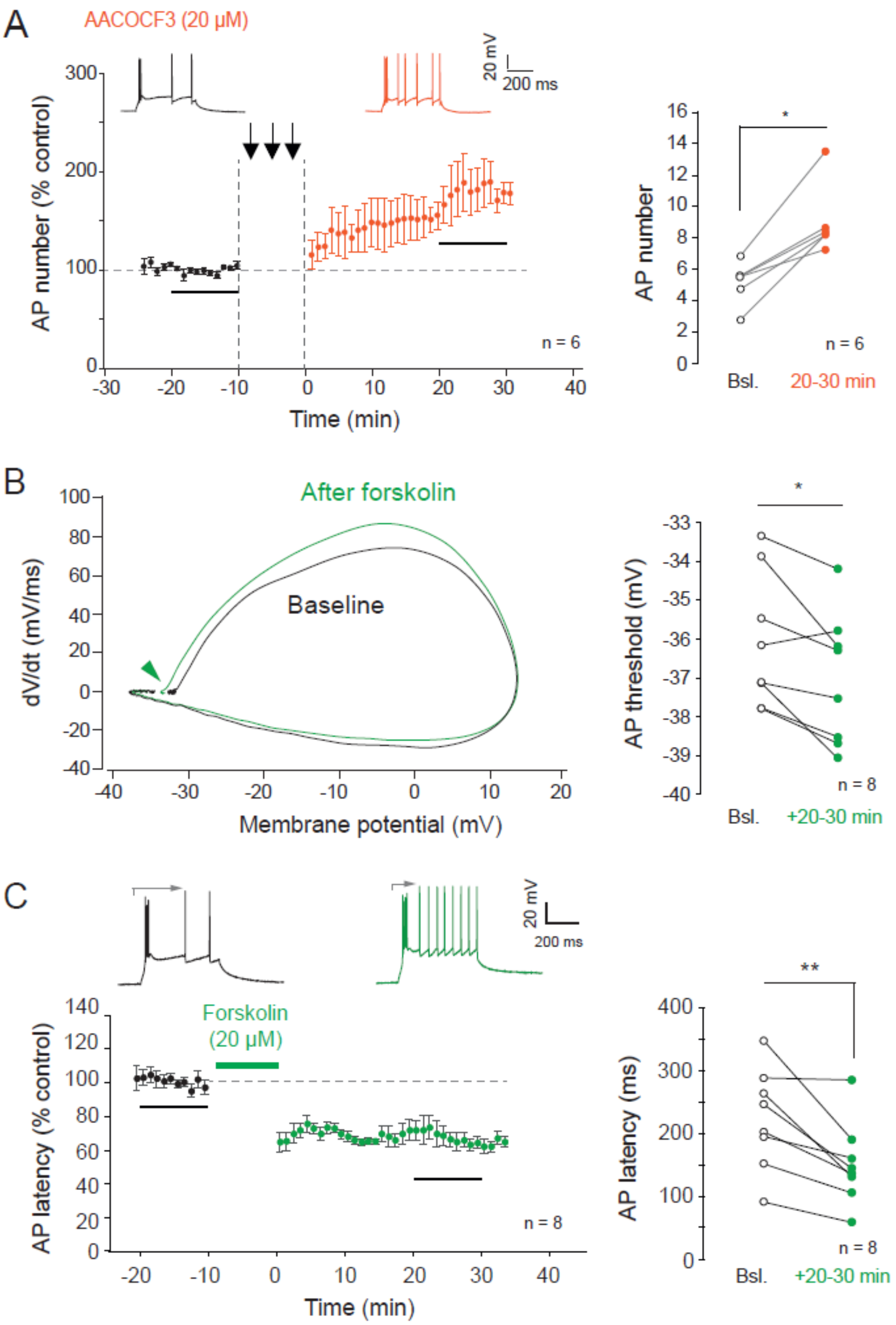

Fig. S8. Induction of LTP-IE in the presence of an inhibitor of AA production and changes in spike threshold and latency following application of forskolin.
A. Induction of LTP-IE in dLGN neurons is not blocked by the inhibitor of arachidonic acid synthesis, AACOCF3. Left, time course. Right, pooled data. Wilcoxon test, *, $p<0.05$. B. AP threshold hyperpolarization following forskolin application. Left, phase plots. Right, pooled data. Wilcoxon, *, $p<0.05$. C. Reduction of spike latency following application of forskolin. Left, time course. Right, group data. Wilcoxon test, **, $p<0.01$.

REFERENCES AND NOTES

1. T. N. Wiesel, D. H. Hubel, Effects of visual deprivation on morphology and physiology of cells in the cats lateral geniculate body. *J. Neurophysiol.* **26**, 978–993 (1963).

2. S. M. Sherman, The thalamus is more than just a relay. *Curr. Opin. Neurobiol.* **17**, 417–422 (2007).

3. T. Rose, T. Bonhoeffer, Experience-dependent plasticity in the lateral geniculate nucleus. *Curr. Opin. Neurobiol.* **53**, 22–28 (2018).

4. M. Duménieu, B. Marquèze-Pouey, M. Russier, D. Debanne, Mechanisms of plasticity in subcortical visual areas. *Cells* **10**, 3162 (2021).

5. H. Ikeda, M. J. Wright, Properties of LGN cells in kittens reared with convergent squint: A neurophysiological demonstration of amblyopia. *Exp. Brain Res.* **25**, 63–77 (1976).

6. R. F. Hess, B. Thompson, G. Gole, K. T. Mullen, Deficient responses from the lateral geniculate nucleus in humans with amblyopia. *Eur. J. Neurosci.* **29**, 1064–1070 (2009).

7. S. Hammer, A. Monavarfeshani, T. Lemon, J. Su, M. A. Fox, Multiple retinal axons converge onto relay cells in the adult mouse thalamus. *Cell Rep.* **12**, 1575–1583 (2015).

8. J. L. Morgan, D. R. Berger, A. W. Wetzel, J. W. Lichtman, The fuzzy logic of network connectivity in mouse visual thalamus. *Cell* **165**, 192–206 (2016).

9. S. B. Rompani, F. E. Müllner, A. Wanner, C. Zhang, C. N. Roth, K. Yonehara, B. Roska, Different modes of visual integration in the lateral geniculate nucleus revealed by single-cell-initiated transsynaptic tracing. *Neuron* **93**, 767–776.e6 (2017).

10. J.-P. Sommeijer, M. Ahmadlou, M. H. Saiepour, K. Seignette, R. Min, J. A. Heimel, C. N. Levelt, Thalamic inhibition regulates critical-period plasticity in visual cortex and thalamus. *Nat. Neurosci.* **20**, 1715–1721 (2017).

11. J. Jaepel, M. Hübener, T. Bonhoeffer, T. Rose, Lateral geniculate neurons projecting to primary visual cortex show ocular dominance plasticity in adult mice. *Nat. Neurosci.* **20**, 1708–1714 (2017).

12. R. Mozzachiodi, J. H. Byrne, More than synaptic plasticity: Role of nonsynaptic plasticity in learning and memory. *Trends Neurosci.* **33**, 17–26 (2010).

13. D. Debanne, Y. Inglebert, M. Russier, Plasticity of intrinsic neuronal excitability. *Curr. Opin. Neurobiol.* **54**, 73–82 (2019).

14. V. Sourdet, M. Russier, G. Daoudal, N. Ankri, D. Debanne, Long-term enhancement of neuronal excitability and temporal fidelity mediated by metabotropic glutamate receptor subtype 5. *J. Neurosci.* **23**, 10238–10248 (2003).

15. R. H. Cudmore, G. G. Turrigiano, Long-term potentiation of intrinsic excitability in LV visual cortical neurons. *J. Neurophysiol.* **92**, 341–348 (2004).

16. C. D. Aizenman, C. J. Akerman, K. R. Jensen, H. T. Cline, Visually driven regulation of intrinsic neuronal excitability improves stimulus detection in vivo. *Neuron* **39**, 831–842 (2003).

17. A. P. Y. Brown, L. Cossell, T. W. Margrie, Visual experience regulates the intrinsic excitability of visual cortical neurons to maintain sensory function. *Cell Rep.* **27**, 685–689.e4 (2019).

18. K. Nataraj, N. Le Roux, M. Nahmani, S. Lefort, G. Turrigiano, Visual deprivation suppresses L5 pyramidal neuron excitability by preventing the induction of intrinsic plasticity. *Neuron* **68**, 750–762 (2010).

19. S. Suzuki, M. A. Rogawski, T-type calcium channels mediate the transition between tonic and phasic firing in thalamic neurons. *Proc. Natl. Acad. Sci. U.S.A.* **86**, 7228–7232 (1989).

20. V. Crunelli, S. Lightowler, C. E. Pollard, A T-type Ca2+ current underlies low-threshold Ca2+ potentials in cells of the cat and rat lateral geniculate nucleus. *J. Physiol.* **413**, 543–561 (1989).

21. R. H. Cudmore, L. Fronzaroli-Molinieres, P. Giraud, D. Debanne, Spike-time precision and network synchrony are controlled by the homeostatic regulation of the D-type potassium current. *J. Neurosci.* **30**, 12885–12895 (2010).

22. E. M. Goldberg, B. D. Clark, E. Zagha, M. Nahmani, A. Erisir, B. Rudy, K+ channels at the axon initial segment dampen near-threshold excitability of neocortical fast-spiking GABAergic interneurons. *Neuron* **58**, 387–400 (2008).

23. E. Campanac, C. Gasselin, A. Baude, S. Rama, N. Ankri, D. Debanne, Enhanced intrinsic excitability in basket cells maintains excitatory-inhibitory balance in hippocampal circuits. *Neuron* **77**, 712–722 (2013).

24. D. A. McCormick, J. R. Huguenard, A model of the electrophysiological properties of thalamocortical relay neurons. *J. Neurophysiol.* **68**, 1384–1400 (1992).

25. M. H. P. Kole, J. J. Letzkus, G. J. Stuart, Axon initial segment Kv1 channels control axonal action potential waveform and synaptic efficacy. *Neuron* **55**, 633–647 (2007).

26. S. Goethals, R. Brette, Theoretical relation between axon initial segment geometry and excitability. *eLife* **9**, e53432 (2020).

27. D. Oliver, C.-C. Lien, M. Soom, T. Baukrowitz, P. Jonas, B. Fakler, Functional conversion between A-type and delayed rectifier K+ channels by membrane lipids. *Science* **304**, 265–270 (2004).

28. M. Carta, F. Lanore, N. Rebola, Z. Szabo, S. V. Da Silva, J. Lourenço, A. Verraes, A. Nadler, C. Schultz, C. Blanchet, C. Mulle, Membrane lipids tune synaptic transmission by direct modulation of presynaptic potassium channels. *Neuron* **81**, 787–799 (2014).

29. M.-F. Fong, P. S. Finnie, T. Kim, A. Thomazeau, E. S. Kaplan, S. F. Cooke, M. F. Bear, Distinct laminar requirements for NMDA receptors in experience-dependent visual cortical plasticity. *Cereb. Cortex* **30**, 2555–2572 (2020).

30. S. M. Sherman, Thalamus plays a central role in ongoing cortical functioning. *Nat. Neurosci.* **19**, 533–541 (2016).

31. D. M. Blitz, W. G. Regehr, Retinogeniculate synaptic properties controlling spike number and timing in relay neurons. *J. Neurophysiol.* **90**, 2438–2450 (2003).

32. R. Pigeat, P. Chausson, F. M. Dreyfus, N. Leresche, R. C. Lambert, Sleep slow wave-related homo and heterosynaptic LTD of intrathalamic GABAAergic synapses: Involvement of T-type Ca2+ channels and metabotropic glutamate receptors. *J. Neurosci.* **35**, 64–73 (2015).

33. N. Leresche, R. C. Lambert, T-type calcium channels in synaptic plasticity. *Channels (Austin)* **11**, 121–139 (2017).

34. J. Ziburkus, E. K. Dilger, F.-S. Lo, W. Guido, LTD and LTP at the developing retinogeniculate synapse. *J. Neurophysiol.* **102**, 3082–3090 (2009).

35. M. D. Evans, R. P. Sammons, S. Lebron, A. S. Dumitrescu, T. B. K. Watkins, V. N. Uebele, J. J. Renger, M. S. Grubb, Calcineurin signaling mediates activity-dependent relocation of the axon initial segment. *J. Neurosci.* **33**, 6950–6963 (2013).

36. E. K. Dilger, T. E. Krahe, D. R. Morhardt, T. A. Seabrook, H.-S. Shin, W. Guido, Absence of plateau potentials in dLGN cells leads to a breakdown in retinogeniculate refinement. *J. Neurosci.* **35**, 3652–3662 (2015).

37. D. J. Denman, D. Contreras, On parallel streams through the mouse dorsal lateral geniculate nucleus. *Front. Neural Circuits* **10**, 20 (2016).

38. B. Sriram, P. M. Meier, P. Reinagel, Temporal and spatial tuning of dorsal lateral geniculate nucleus neurons in unanesthetized rats. *J. Neurophysiol.* **115**, 2658–2671 (2016).

39. C. M. Gray, W. Singer, Stimulus-specific neuronal oscillations in orientation columns of cat visual cortex. *Proc. Natl. Acad. Sci. U.S.A.* **86**, 1698–1702 (1989).

40. W. Singer, C. M. Gray, Visual feature integration and the temporal correlation hypothesis. *Annu. Rev. Neurosci.* **18**, 555–586 (1995).

41. C. Pedroarena, R. Llinás, Dendritic calcium conductances generate high-frequency oscillation in thalamocortical neurons. *Proc. Natl. Acad. Sci. U.S.A.* **94**, 724–728 (1997).

42. R. Llinás, U. Ribary, D. Contreras, C. Pedroarena, The neuronal basis for consciousness. *Philos. Trans. R. Soc. Lond. B Biol. Sci.* **353**, 1841–1849 (1998).

43. R. A. W. Galuske, M. H. J. Munk, W. Singer, Relation between gamma oscillations and neuronal plasticity in the visual cortex. *Proc. Natl. Acad. Sci. U.S.A.* **116**, 23317–23325 (2019).

44. E. Campanac, G. Daoudal, N. Ankri, D. Debanne, Downregulation of dendritic I(h) in CA1 pyramidal neurons after LTP. *J. Neurosci.* **28**, 8635–8643 (2008).

45. S. Incontro, M. Sammari, F. Azzaz, Y. Inglebert, N. Ankri, M. Russier, J. Fantini, D. Debanne, Endocannabinoids tune intrinsic excitability in O-LM interneurons by direct modulation of Postsynaptic Kv7 channels. *J. Neurosci.* **41**, 9521–9538 (2021).

46. M. Sammari, Y. Inglebert, N. Ankri, M. Russier, S. Incontro, D. Debanne, Theta patterns of stimulation induce synaptic and intrinsic potentiation in O-LM interneurons. *Proc. Natl. Acad. Sci. U.S.A.* **119**, e2205264119 (2022).

47. M. R. Williams, J. R. Fuchs, J. T. Green, A. D. Morielli, Cellular mechanisms and behavioral consequences of Kv1.2 regulation in the rat cerebellum. *J. Neurosci.* **32**, 9228–9237 (2012).

48. K. Funke, H. C. Pape, U. T. Eysel, Noradrenergic modulation of retinogeniculate transmission in the cat. *J. Physiol.* **463**, 169–191 (1993).

49. M. S. Grubb, F. M. Rossi, J. P. Changeux, I. D. Thompson, Abnormal functional organization in the dorsal lateral geniculate nucleus of mice lacking the beta 2 subunit of the nicotinic acetylcholine receptor. *Neuron* **40**, 1161–1172 (2003).

50. M. S. Grubb, I. D. Thompson, Visual response properties in the dorsal lateral geniculate nucleus of mice lacking the beta2 subunit of the nicotinic acetylcholine receptor. *J. Neurosci.* **24**, 8459–8469 (2004).

51. P. Orlowska-Feuer, M. K. Smyk, K. Palus-Chramiec, K. Dyl, M. H. Lewandowski, Orexin A as a modulator of dorsal lateral geniculate neuronal activity: A comprehensive electrophysiological study on adult rats. *Sci. Rep.* **9**, 16729 (2019).

52. G. Sokhadze, K. L. Whyland, M. E. Bickford, W. Guido, The organization of cholinergic projections in the visual thalamus of the mouse. *J. Comp. Neurol.* **530**, 1081–1098 (2022).

53. J. D. S. Reggiani, Q. Jiang, M. Barbini, A. Lutas, L. Liang, J. Fernando, F. Deng, J. Wan, Y. Li, C. Chen, M. L. Andermann, Brainstem serotonin neurons selectively gate retinal information flow to thalamus. *Neuron* **111**, 711–726.e11 (2023).

54. N. Li, Q. Liu, Y. Zhang, Z. Yang, X. Shi, Y. Gu, Cortical feedback modulates distinct critical period development in mouse visual thalamus. *iScience* **26**, 105752 (2023).

55. D. A. McCormick, T. Bal, Sleep and arousal: Thalamocortical mechanisms. *Annu. Rev. Neurosci.* **20**, 185–215 (1997).

56. M. Zbili, S. Rama, P. Yger, Y. Inglebert, N. Boumedine-Guignon, L. Fronzaroli-Moliniere, R. Brette, M. Russier, D. Debanne, Axonal Na+ channels detect and transmit levels of input synchrony in local brain circuits. *Sci. Adv.* **6**, eaay4313 (2020).

57. G. E. Little, G. López-Bendito, A. E. Rünker, N. García, M. C. Piñon, A. Chédotal, Z. Molnár, K. J. Mitchell, Specificity and plasticity of thalamocortical connections in Sema6A mutant mice. *PLOS Biol.* **7**, e98 (2009).

58. M. Zbili, D. Debanne, Myelination increases the spatial extent of analog-digital modulation of synaptic transmission: A modeling study. *Front. Cell. Neurosci.* **14**, 40 (2020).

59. M. Zbili, S. Rama, M.-J. Benitez, L. Fronzaroli-Molinieres, A. Bialowas, N. Boumedine-Guignon, J. J. Garrido, D. Debanne, Homeostatic regulation of axonal Kv1.1 channels accounts for both synaptic and intrinsic modifications in the hippocampal CA3 circuit. *Proc. Natl. Acad. Sci. U.S.A.* **118**, e2110601118 (2021).

60. J. Ziburkus, W. Guido, Loss of binocular responses and reduced retinal convergence during the period of retinogeniculate axon segregation. *J. Neurophysiol.* **96**, 2775–2784 (2006).

61. J. Bauer, S. Weiler, M. H. P. Fernholz, D. Laubender, V. Scheuss, M. Hübener, T. Bonhoeffer, T. Rose, Limited functional convergence of eye-specific inputs in the retinogeniculate pathway of the mouse. *Neuron* **109**, 2457–2468.e12 (2021).

62. B. M. Hooks, C. Chen, Distinct roles for spontaneous and visual activity in remodeling of the retinogeniculate synapse. *Neuron* **52**, 281–291 (2006).

63. T. E. Krahe, W. Guido, Homeostatic plasticity in the visual thalamus by monocular deprivation. *J. Neurosci.* **31**, 6842–6849 (2011).

64. T. G. Weyand, M. Boudreaux, W. Guido, Burst and tonic response modes in thalamic neurons during sleep and wakefulness. *J. Neurophysiol.* **85**, 1107–1118 (2001).

65. M. Stimberg, R. Brette, D. F. Goodman, Brian 2, an intuitive and efficient neural simulator. *eLife* **8**, e47314 (2019).

66. S. G. Birnbaum, A. W. Varga, L.-L. Yuan, A. E. Anderson, J. D. Sweatt, L. A. Schrader, Structure and function of Kv4-family transient potassium channels. *Physiol. Rev.* **84**, 803–833 (2004).

67. Y. Carrasquillo, J. M. Nerbonne, IA channels: Diverse regulatory mechanisms. *Neuroscientist* **20**, 104–111 (2014).

68. A. Lorincz, Z. Nusser, Cell-type-dependent molecular composition of the axon initial segment. *J. Neurosci.* **28**, 14329–14340 (2008).

69. S. G. Meuth, T. Kanyshkova, P. Meuth, P. Landgraf, T. Munsch, A. Ludwig, F. Hofmann, H.-C. Pape, T. Budde, Membrane resting potential of thalamocortical relay neurons is shaped by the interaction among TASK3 and HCN2 channels. *J. Neurophysiol.* **96**, 1517–1529 (2006).

70. A. Teska, M. Stimberg, R. Brette, brian2modelfitting, version 0.4 (Zenodo, 2020); https://doi.org/10.5281/zenodo.4601961.

71. S. Druckmann, Y. Banitt, A. Gidon, F. Schürmann, H. Markram, I. Segev, A novel multiple objective optimization framework for constraining conductance-based neuron models by experimental data. *Front. Neurosci.* **1**, 7–18 (2007).

72. R. M. Storn, K. Price, Differential evolution—A simple and efficient heuristic for global optimization over continuous spaces. *J. Global Optim.* **11**, 341–359 (1997).

73. L. Naudin, N. Corson, M. A. Aziz-Alaoui, J. L. Jiménez Laredo, T. Démare, On the modeling of the three types of non-spiking neurons of the caenorhabditis elegans. *Int. J. Neural Syst.* **31**, 2050063 (2021).

74. L. Naudin, J. L. Jiménez Laredo, Q. Liu, N. Corson, Systematic generation of biophysically detailed models with generalization capability for non-spiking neurons. *PLOS ONE* **17**, e0268380 (2022).

75. L. Buhry, S. Saighi, A. Giremus, E. Grivel, S. Renaud, "Parameter estimation of the Hodgkin-Huxley model using metaheuristics: Application to neuromimetic analog integrated circuits" in *2008 IEEE Biomedical Circuits and Systems Conference* (IEEE, 2008), pp. 173–176.

76. J. R. Huguenard, D. A. McCormick, Simulation of the currents involved in rhythmic oscillations in thalamic relay neurons. *J. Neurophysiol.* **68**, 1373–1383 (1992).

77. J. R. Huguenard, D. A. Prince, Slow inactivation of a TEA-sensitive K current in acutely isolated rat thalamic relay neurons. *J. Neurophysiol.* **66**, 1316–1328 (1991).
